# Enhanced optical and electrochemical properties MoO_3_-NiO-NiMoO_4_ ternary nanocomposite thin films: influence of PEG and PVA additives

**DOI:** 10.1038/s41598-025-02911-1

**Published:** 2025-07-24

**Authors:** F. Aghaei, F. E. Ghodsi, J. Mazloom

**Affiliations:** Department of Physics, Faculty of Science, University of Guilan, Namjoo Avenue, P.O.Box 413351914, Rasht, Iran

**Keywords:** MoO_3_-NiO-NiMoO_4_ composite, Spin-coating, Polymer effect, Optical constant, Electrochemical activity, Applied physics, Chemical physics

## Abstract

In this study, a nanocomposite consisting of Molybdenum trioxide, nickel oxide, and nickel molybdate (MoO_3_-NiO-NiMoO_4_) was synthesized by the spin coating technique of sol–gel. We investigated the impact of polyethylene glycol (PEG) and polyvinylalcohol (PVA) on the physical properties of the nanocomposite through various analytical methods. XRD analysis revealed a composite structure consisting of the orthorhombic stable phase of MoO_3_, the cubic phase of NiO, and the monoclinic of NiMoO_4_ with an average crystallite size of between 39 and 58 nm. Furthermore, FESEM and AFM images indicated that surface morphology and roughness parameters changed dramatically with adding polymers. Interestingly, adding PEG and PVA polymers decreased refractive indices and extinction coefficients of prepared thin films, while the values of the band gap improved to 3.89 eV by adding PEG. Based on CV and GCD results, we found that PVA addition significantly enhanced the electrochemical performance of MoO_3_-NiO-NiMoO_4_ thin films, whereas PEG addition had minimal effects. The MoO_3_-NiO-NiMoO_4_/PVA nanocomposite exhibited the best performance at a current density of 0.1 mA cm^−2^, with a specific capacity of 44.13 mF cm^-2^. Moreover, MoO_3_-NiO-NiMoO_4_/PVA demonstrated a high capacity retention rate at a current density of 1 mA cm^−2^, indicating a stability of 97.36% over 5000 charge/discharge cycles.

## Introduction

For nearly two centuries, fossil fuels have served as the primary means of powering human civilization, resulting in the depletion of energy resources and substantial environmental harm. Consequently, the global energy crisis is a crucial issue requiring attention worldwide. Pursuing an affordable, sustainable, and renewable energy source lies at the forefront of contemporary challenges^1^. Transitional metal oxides (TMOs) represent a distinct and abundant material class known for their non-toxicity, thermal stability, and the ability to exhibit favorable electrical conductivity and catalytic activity. Over the past fifty years, extensive research has been dedicated to studying TMO materials due to their unique chemistry and distinguished physical properties, primarily deriving from their partially filled d orbitals. Within this category exists a group of TMOs recognized for their distinctive layered arrangement and diverse oxidation states. Examples include V_2_O_5_, NiO, MnO_2_, MoO_3_, and WO_3_^2^. The layered structures feature open channels that facilitate the injection and extraction of ions, making these TMOs dependable options for various devices of semiconductors, ionic batteries, energy storage device^3^, particularly in sensors^4^, filters^5^, de-icing^6^, electrochromic and photochromic applications. However, insufficient electrical conductivity and size-related stability challenges reduce specific capacitance, limiting practical commercialization. Addressing these critical issues and enhancing the kinetics of ion/electron transport involves the implementation of various modification techniques such as doping^7^, conductive wrapping^8^, and heterostructured nanocomposites^9,10^. Several studies have showcased the potential of different transition metal oxide composites (TMOCs) for energy storage applications^11–13^.

Molybdenum trioxide (MoO_3_) has received less attention in research than other metal oxides. Consequently, there exists ample opportunity to delve deeper into exploring the potential of this material. MoO_3_ is a widely recognized oxide with significant applications across various technological domains, including energy storage^14^, catalysis^15^, solution-processable solar cells^16^, electrochromics^17^, photochromics^18^, thermochromics^19^, sensors^20^, and lubricants^21^.

Crystalline MoO_3_ exhibits three polymorphs: orthorhombic stable phase from the thermodynamic point of view (α-MoO_3_), along with two met-stable phases, hexagonal (h-MoO_3_) and monoclinic (β-MoO_3_). Hence, there has been a growing emphasis on the targeted fabrication of MoO_3_ nanoparticles with distinct morphologies and appealing material characteristics for years. Although various Mo-O crystal structures have been investigated in the literature, a more comprehensive understanding of the synthesis chemistry of MoO_3_ and associated structure data is needed. Intriguingly, the formation reaction conditions predominantly support the growth of the stable phase of α-MoO_3_ alone.

Recently, there has been a notable interest in using metal oxide semiconductors to develop composites in various applications. Among these, nickel oxide (NiO) is a semitransparent p-type semiconductor with a significant direct gap (3.6–3.9 eV)^22^. NiO adopts a face-centered cubic structure^23^. NiO thin films demonstrate exceptional thermal and chemical stability, along with outstanding electrical, optical, magnetic, electrochromic, and electrochemical characteristics^24^.

Metal molybdates are well-known mixed-metal oxides due to their advantageous electronic setup, stable crystal structure, redox properties, remarkable physical/chemical traits, and relatively strong electronic conductivity^25^. In particular, NiMoO_4_ stands out as a superior catalyst in various industrial processes, boasting higher catalytic activity compared to other molybdates, attributed to its denser oxidation states close to the upper edge of the valence band^26^. While NiMoO_4_ has been utilized since the mid-twentieth century, recent focus has shifted towards nanoscale production due to its potential in electrochemical storage applications, sparking renewed interest^27^.

Polymer nanocomposites are solid materials with multiple phases, wherein at least one component incorporates particles at the nano-scale (ranging from 1 to 100 nm). Certain polymers can modify the porosity of electrode surfaces, thereby enhancing their electrochemical performance^28^. Researches indicate that PVA and PEG exhibit significant influence on the porosity of thin films prepared via sol–gel methods^29,30^. Upon annealing, PEG and PVA undergo decomposition, releasing carbon dioxide and consequently creating pores through gas elimination from the coatings.

This study presents a novel exploration of combining molybdenum trioxide (MoO_3_) with nickel oxide (NiO) and nickel molybdate (NiMoO_4_) using a spin-coating sol–gel technique. While extensive research has been conducted on individual metal oxides and their composites, this work is distinguished by its focus on a less-studied combination of these materials and the innovative application of polyethylene glycol (PEG) and polyvinyl alcohol (PVA) as polymeric modifiers. The structural properties, optical constants, wettability, and electrochemical behavior of thin films are assessed through relevant analysis. The integration of these components in a single composite system and the evaluation of their combined effects on material properties represent a significant departure from conventional approaches. This research addresses gaps in the understanding of MoO_3_ and its composites, offering new insights into their synthesis and potential applications. The unique combination of MoO_3_, NiO, and NiMoO_4_, along with the novel use of polymers in the synthesis process, provides fresh perspectives and contributes to the advancement of material science and energy storage technologies.

## Experimental

### Materials

Nickel (II) nitrate hexahydrate (Ni(NO_3_)_2_.6H_2_O), Ammonium Heptamolybdate tetra-hydrate (AHM) ((NH4)_6_Mo_7_O_24_.4H_2_O), hydrochloric acid (HCl, 37%), polyethylene glycol (PEG, 6000 (H (C_2_H_4_O)_n_ OH)), poly (vinyl alcohol) (PVA, 72000) [(C_2_H_3_(OH)]_n_, potassium hydroxide (KOH) were obtained from Merck Company (Germany). Distilled water (H_2_O) and Fluorine doped tin oxide coated glass (FTO, 7Ω/sq) were purchased from Khazar Ghatran, Iran and Solaronix, Switzerland.

### Thin film preparation

To prepare MoO_3_ thin films, 0.1 M of AHM was dissolved in distilled water (50 mL), which was constantly stirred at 80 °C for 1 h. Small quantities of Concentrated hydrochloric acid (HCl) were introduced into the solution. The pH of the solution obtained was 2.5. This solution was named Sol1 and was used to make other solutions. Also, to prepare NiO thin films, 0.1 M of Nickel (II) nitrate was dissolved in distilled water (50 mL), which was constantly stirred at room temperature for 1 h (Sol2). The sol1 and sol2 were mixed with a volume ratio of 1:1, This solution was named Sol3. A specified amount of PEG (0.6 g by 10 ml) was gradually added to Sol3. To obtain a homogeneous solution, it was stirred at 80 °C for 1 h (Sol4). Also, 0.6 g of PVA was added to 10 mL of the Sol3 and stirred at 80 °C for 1 h (Sol5).

Thin films were made utilizing the spin coating technique of sol–gel on glass and FTO substrates in the atmosphere. The deposition was performed at a rotation speed of 3000 rpm for 20 s, and after each coating process, they were dried at 200 °C for 15 min to remove water and other residual materials. This procedure was iterated five times for every sample. Finally, the films were annealed at 550 °C for 150 min under air atmosphere to improve crystallization. The thin films obtained from solutions Sol1-5 were named M, N, M-N-NM, M-N-NM/PEG, and M-N-NM/PVA, respectively. The chemical formation of MoO_3_ from the precursor solution is obtained as the following equilibrium reaction^31^:

1 $$\left( {{\text{NH}}_{{4}} } \right)_{{6}} {\text{Mo}}_{{7}} {\text{O}}_{{{24}}} .{\text{ 4H}}_{{2}} {\text{O }} + {\text{ 2H}}_{{2}} {\text{O }} \to {\text{ 7MoO}}_{{3}} \left( {\text{s}} \right) \, + {\text{ 6NH}}_{{3}} \left( {\text{g}} \right) + {\text{ 9H}}_{{2}} {\text{O }}\left( {\text{g}} \right)$$

where *s* is the solid state and *g* is the gas state of resultant products.

The incorporation of PEG and PVA in the sol–gel process was strategically aimed at tailoring the porosity, morphology, and electrochemical behavior of the MoO_3_–NiO–NiMoO_4_ thin films. PEG, due to its nonionic and hydrophilic nature, facilitates the formation of a more porous and uniform film upon thermal decomposition, which increases the electroactive surface area^32^. PVA serves as both a binder and a pore-forming agent, influencing the mechanical stability and enhancing the ion transport channels within the film^33^. The selected concentrations were optimized based on preliminary experiments to avoid film cracking while maximizing electrochemical performance. These roles are well-supported by prior reports highlighting the positive impact of such polymers on charge storage efficiency and structural control in oxide-based thin films.

**Fig. 1:**
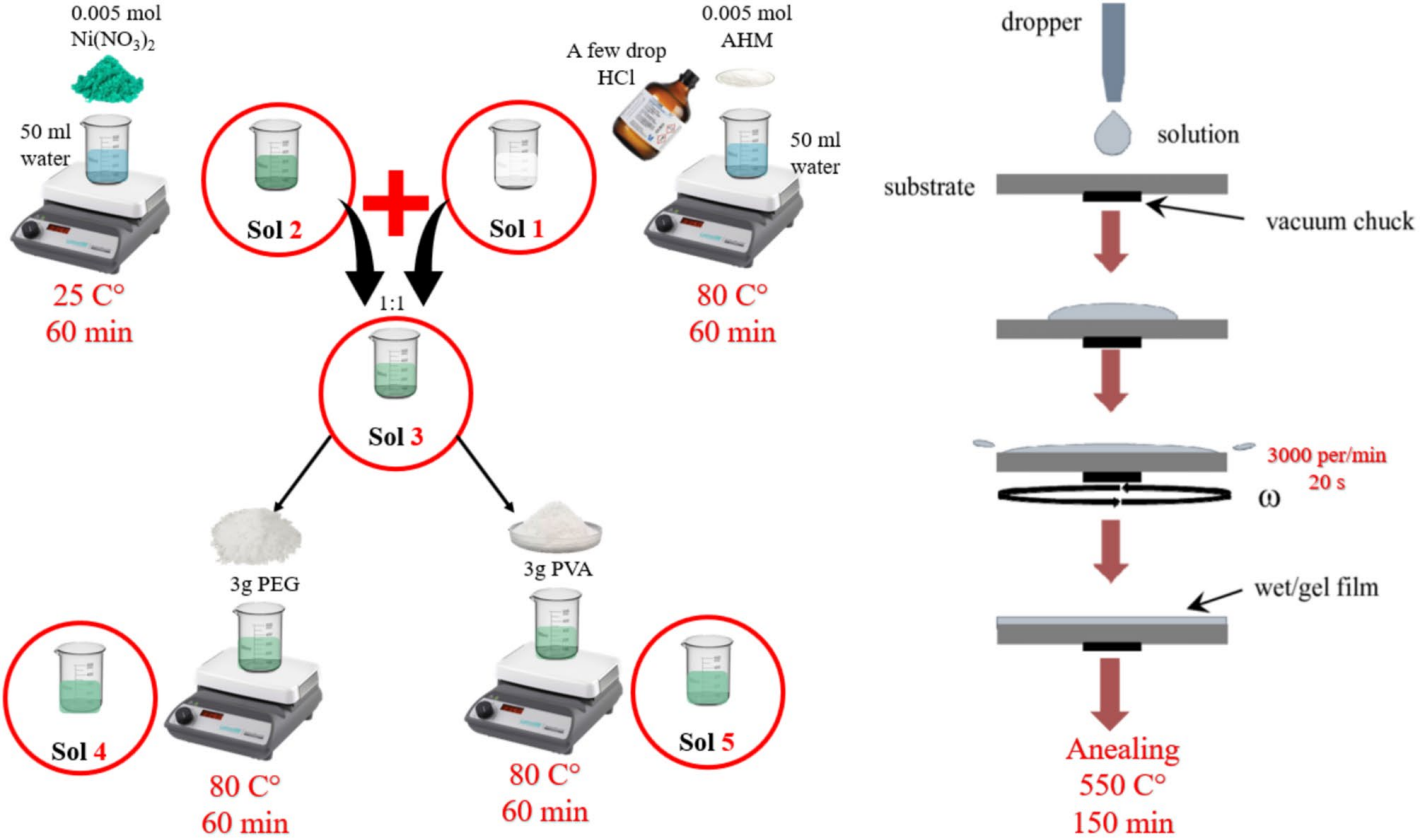
Schematic illustration of the sol–gel spin-coating process used for the fabrication of thin films.

Figure 1 illustrates the schematic representation of the sol–gel spin-coating process used for preparing MoO_3_–NiO–NiMoO_4_-based thin films, incorporating optional polymer additives (PEG or PVA) to tailor structural and electrochemical properties.

### Characterization

The structure properties of the synthesized thin films were checked by the X-ray diffraction (XRD) technique (by X’Pert Pro, Panalytical, Netherland) with wavelength 1.5406 Å from CuKa radiation at room temperature. Step scans were conducted over a 2*θ* angular range of 10° to 80° at a scanning speed of 30 min^-1^. Functional groups and the composition of the synthesized sample were revealed through Fourier transform infrared spectrometer (FT-IR) measurements using an FTIR instrument (8900, Shimadzu, Japan). Energy-dispersive X-ray spectroscopy (EDX) was exploited, utilizing the EDX instrument (EM8000F, KY KY, China), to validate the distribution of constituent elements in the samples. For selecting the optimal composite, the morphology of the thin film surface was exposed through field emission scanning electron microscopy (TESCAN MIRA3, TESCAN, Czech Republic). The roughness parameter was computed utilizing scanning probe electron microscopy (DualScope C-26, DME, USA) and Gwyddion software package. The optical characteristics of thin films prepared through spin coating were examined using an ultraviolet–visible spectrophotometer (Carry 100, Varian, Australia) and diffuse reflectance spectrophotometer DRS (S-4100, Scinco, South Korea). The electrochemical behavior of the samples was studied using a three-electrode cell system (ZIVE SP1, WONATECH, South Korea). The electrochemical test of the composite electrodes was checked using a three-electrode cell system (ZIVE SP1, WONATECH, South Korea). The electrochemical assessments were conducted in a KOH (1 M) electrolyte. Within this configuration, the synthesized sample served as the working electrode, an electrode of Ag/AgCl operated as the reference electrode, and a platinum wire fulfilled the role of the counter electrode.

## Result and discussion

### Structural properties

**Fig. 2:**
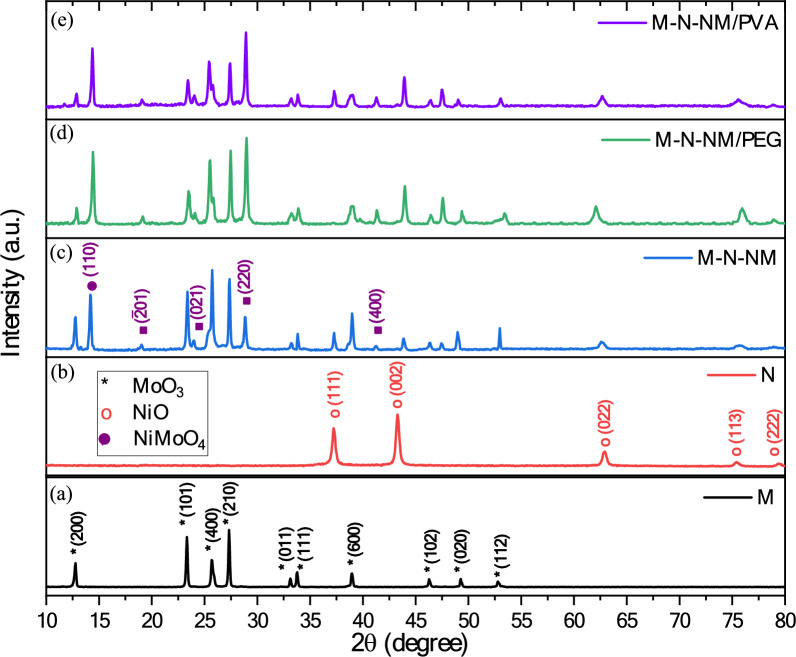
XRD patterns of (**a**) MoO_3_, (**b**) NiO, (**c**) MoO_3_-NiO-NiMoO_4_, (**d**) MoO_3_-NiO-NiMoO_4_/PEG, and (**e**) MoO_3_-NiO-NiMoO_4_/PVA thin films, respectively.

To study the structure of the spin-coated films, we performed the X-ray diffraction (XRD) technique. The XRD patterns for all thin film samples are presented in Fig. 2. All the peaks attributed to pure MoO_3_ nanoparticles align with the orthorhombic structured α-MoO_3_ (JCPDS card no: 01-076-1003). Also, the observed peaks corresponding to the pure NiO phase can be attributed to the cubic structure of NiO (JCPDS card no: 01-075-0197). The XRD profile of the MoO_3_-NiO-NiMoO_4_ composites distinctly indicates the dual-phase nature of the precursor material. Alongside the peaks corresponding to α-MoO_3_, prominent new peaks at 2*θ* = 14.19°, 19.02°, 23.95°, 28.84°, and 41.24° are identified as (110), ($\overline{2 }$ 01), (021), (220) and (400) crystal planes of the monoclinic phase of NiMoO_4_, respectively (JCPDS card no: 00-031-0902).

The average crystallite size (*D*) and strain of samples were obtained by the Williamson-Hall formula^31^:

2 $$\beta cos\left(\theta \right)=\frac{k\lambda }{D} +4\varepsilon sin(\theta )$$

where *θ* is the Bragg diffraction angle,* ε* is the strain,* λ* is the wavelength of the radiation (1.54056 Å for CuKα radiation),* D* is the crystallite size, *K* is a constant equal to 0.94, and *β* is full width at half maximum (FWHM).

The dislocation density (*δ*), and stacking fault (*SF*) values can be obtained using the following relations^34,35^:

3 $$\delta =\frac{1}{{D}^{2}}$$

4 $$SF=\left[\frac{2 {\pi }^{2}}{45{\left(3tan\theta \right)}^\frac{1}{2}}\right]\beta$$

**Fig. 3:**
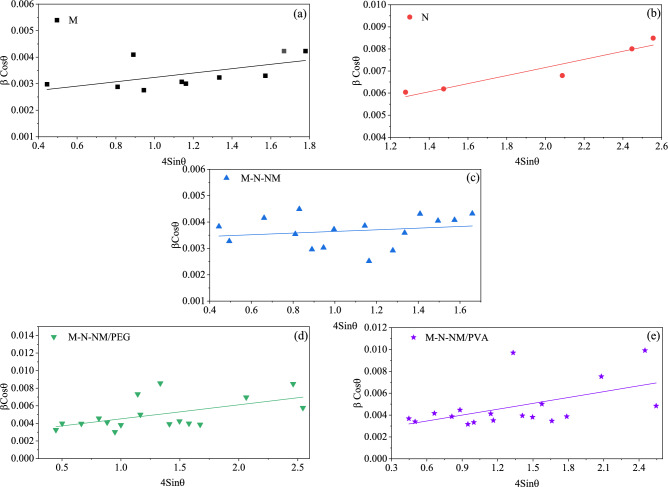
Williamson-Hall plots of (**a**) MoO_3_, (**b**) NiO, (**c**) MoO_3_-NiO-NiMoO_4_, (**d**) MoO_3_-NiO-NiMoO_4_/PEG, and (**e**) MoO_3_-NiO-NiMoO_4_/PVA thin films, respectively.

To calculate the crystallite size and strain of the composites, the graph of β*Cos*θ in terms of 4*Sin*θ at different diffraction angles was drawn in Fig. 3. Due to the linearity of the above formula, the strain of the composites was calculated using the slope and the value of crystallite size from the intercept. The average crystallite size revealed values of 57.5 nm for the M sample, 39.7 nm for the N sample, 41.8 nm for the M-N-NM sample, 47.8 nm for the M-N-NM/PEG composite, and 58.2 nm for the M-N-NM/PVA composite. Concurrently, the strain values were calculated, showing 0.18 for the M sample, 0.28 for the N sample, 0.13 for the M-N-NM sample, 0.26 for the M-N-NM/PEG composite, and 0.27 for the M-N-NM/PVA composite. Also, for each sample, the crystallite size was calculated using the Debye–Scherrer equation based on the most intense peak in the pattern. This value represents the dominant grain size contributing to the crystalline structure of the material. The corresponding 2θ values, crystallographic planes, and calculated crystallite sizes are summarized in Table 1, highlighting the variations in grain size influenced by the incorporation of different additives and synthesis conditions.

**Table 1:** Crystallographic parameters derived from XRD analysis for the synthesized samples.

Sample	*2θ* ( °)	(hkl)	*D* (nm)	*ε* (%)	*δ* (× 10^–5^ nm^−2^)	*D*_*W-H*_ (nm)	*ε*_*W-H*_ (%)	*SF* (× 10^−5^ nm^−1^)	*X*_*c*_
M	23.32	(210)	47.5	0.36	44.3	57.5	0.18	7.61	74.26
N	43.29	(002)	23.8	0.39	176.5	39.7	0.28	16.62	60.15
M-N-NM	25.72	(400)	46.3	0.34	46.6	41.8	0.13	7.25	68.49
M-N-NM/PEG	28.96	(220)	34.3	0.39	84.9	47.8	0.26	11.74	81.64
M-N-NM/PVA	28.91	(220)	40.2	0.35	61.9	58.2	0.27	8.57	61.92

These findings indicate significant variations in both crystallite size and strain across the different samples, suggesting that adding PEG and PVA polymers influences the microstructural properties of the composites. The observed reduction in crystallite size for the M-N-NM and M-N-NM/PEG composites, accompanied by varying strain values (Table 1), highlights the potential impact of polymer incorporation on the mechanical and structural properties of the composites.

To further investigate the structural order of the synthesized nanocomposite thin films, the crystallinity percentage of each sample was calculated based on the XRD patterns. The analysis was carried out using Origin software by deconvoluting the diffraction pattern into crystalline peaks and amorphous background components. Crystalline peaks were fitted using Gaussian functions corresponding to sharp diffraction maxima, while the amorphous content was estimated by integrating the broad hump in the low-intensity background. The crystallinity percentage (*X*_*c*_) was calculated using the following relation^36^:

5 $${X}_{c}=\frac{{A}_{c}}{{A}_{a}+{A}_{c}}\times 100$$

where *A*_*c*_ and *A*_*a*_ represent the integrated areas under the crystalline and amorphous regions, respectively. The calculated crystallinity values percentages of all samples are presented in Table 1. These results indicate that the Mo sample exhibits the highest degree of crystallinity, which is attributed to its high-purity crystalline phase and well-ordered atomic arrangement. In contrast, the lower crystallinity observed in the Ni and Mo-Ni/PVA samples may be due to the presence of disordered or amorphous regions introduced during synthesis. Interestingly, the addition of PEG as a polymeric additive significantly improved the crystallinity compared to PVA. This can be explained by the better templating ability of PEG, which facilitates more uniform nucleation and crystal growth during film formation. Higher crystallinity is often associated with enhanced electronic conductivity and structural stability, both of which are crucial for electrochemical performance in energy storage applications^37^.

### Vibrational spectroscopy

**Fig. 4:**
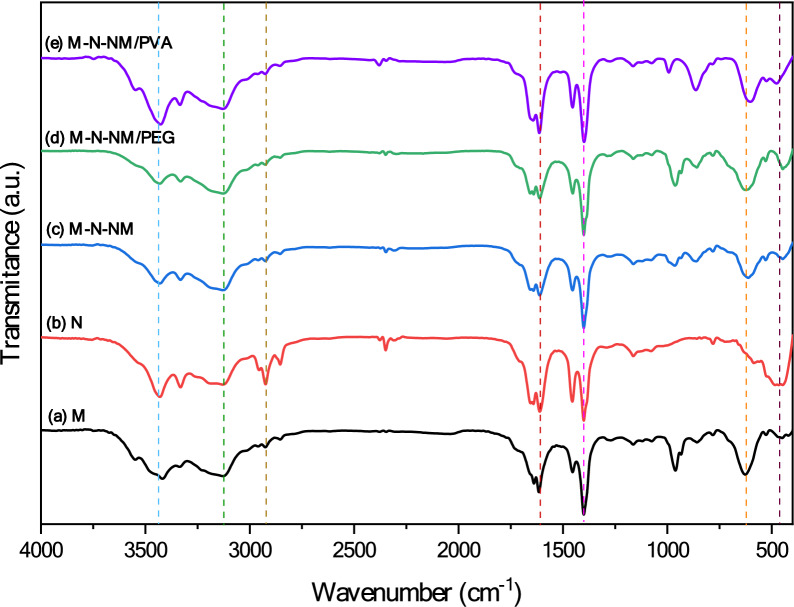
FTIR spectra of MoO_3_, NiO, MoO_3_-NiO-NiMoO_4_, MoO_3_-NiO-NiMoO_4_/PEG, and MoO_3_-NiO-NiMoO_4_/PVA thin films, respectively.

The formation of the phase was additionally validated through XRD analysis and is strongly corroborated by the FTIR findings. The FTIR spectra for MoO_3_ and NiO films and MoO_3_-NiO-NiMoO_4_ composite have been noted from 400 to 4000 cm^−1^, as presented in Fig. 4. In the context of MoO_3_ thin film, illustrated in Fig. 4 displays a broad absorption band at approximately 3427 cm^−1^, which is attributed to the O–H stretching vibrations of physically adsorbed water molecules or hydroxyl groups on the film surface. The peaks at 2380 cm^−1^ appear to originate from the bending mode of HO–H in water representation^26^. Bands at 3126, 1613and 1398 cm^−1^ result from the vibration and stretching bending of N–H in NH^4+^ masses originating from using AHM as the precursor^38^. Bands within the 400–1000 cm^−1^ range indicate the vibration and stretching bending caused by the metal–oxygen bonds^39^. The peak at 993 cm^-1^ shows the stretching of Mo-O, serving as an index for the layer-by-layer phase of α-MoO_3_. The absorption peak at 863 cm^−1^ is attributed to the oxygen stretching state in Mo–O–Mo, while the band at 602 is linked to the bending vibration of Mo–O–Mo^40^.

The FTIR spectra of NiO thin film exhibit a peak approximately at 466 cm^−1^, corresponding to the Ni–O stretching mode^41^. Two absorption peaks around 1611 cm^−1^ and 1400 cm^−1^ are related to the asymmetrical and symmetrical vibrations of the NO^3−^, respectively^42^. The FTIR spectra of MoO_3_-NiO-NiMoO_4_ composites reveal bands at 964 and 862 cm^−1^, attributed to tetrahedra vibration of extremely distorted MoO_4_ in β-NiMoO_4_^43^. While the 447 and 612 cm^−1^ bands in the low wavenumber region indicate the attendance of NiO and MoO_3_ groups in thin films. In the FTIR spectra of the composites, the absorption peak at 1611 cm^−1^ is due to the attendance of NO^3-^ from Ni(NO_3_)_2_ during synthesis, and the absorption peak of about 1400 cm^−1^ corresponds to the bending vibration of the N-H bond in the NH^4+^ group due to the use of AHM^44^. Considering the absence of any additional peaks in M-N-NM/PEG and M-N-NM/PVA samples, the complete elimination of the organic phase due to calcination is evident.

### Elemental analysis

**Fig. 5:**
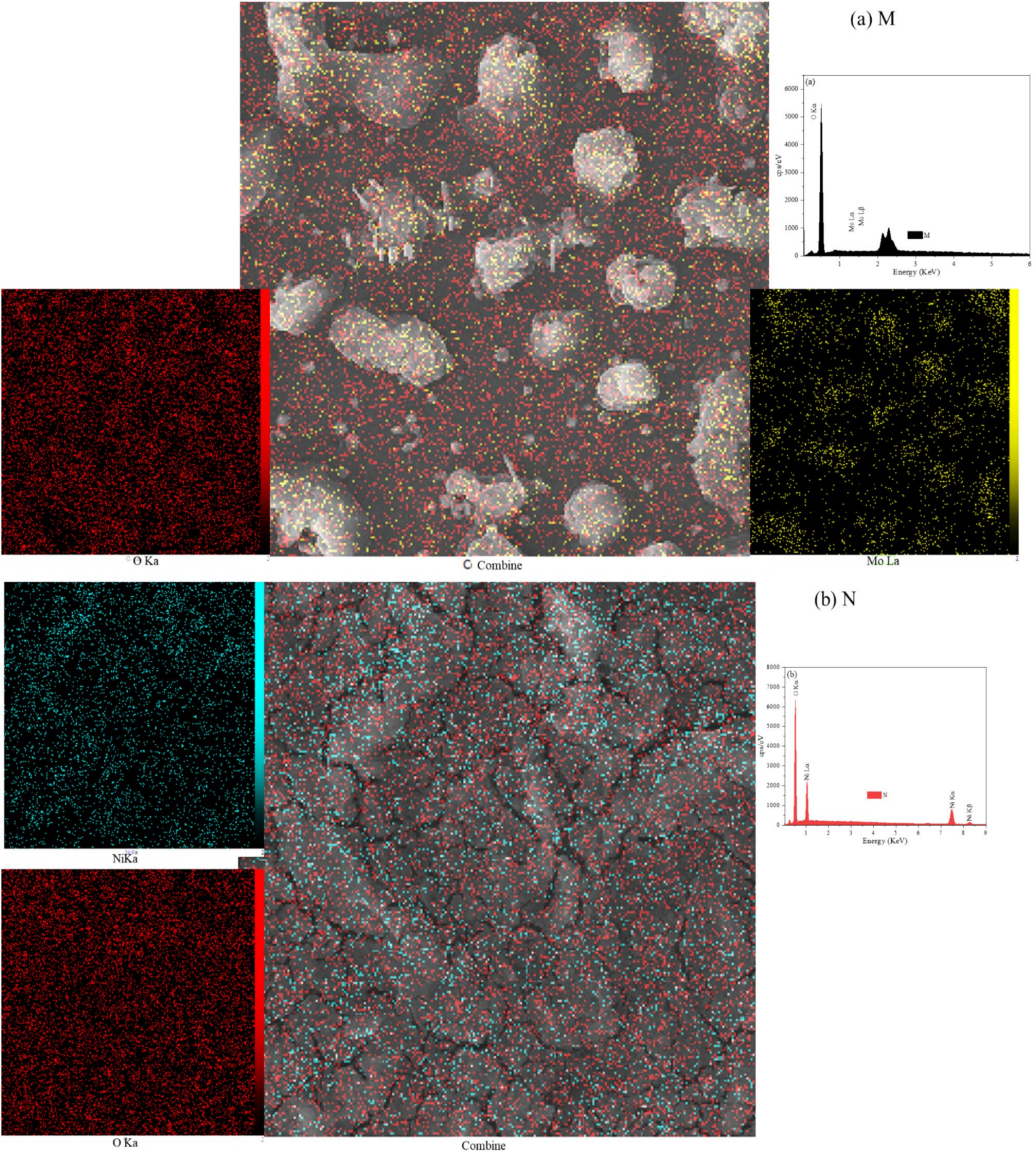

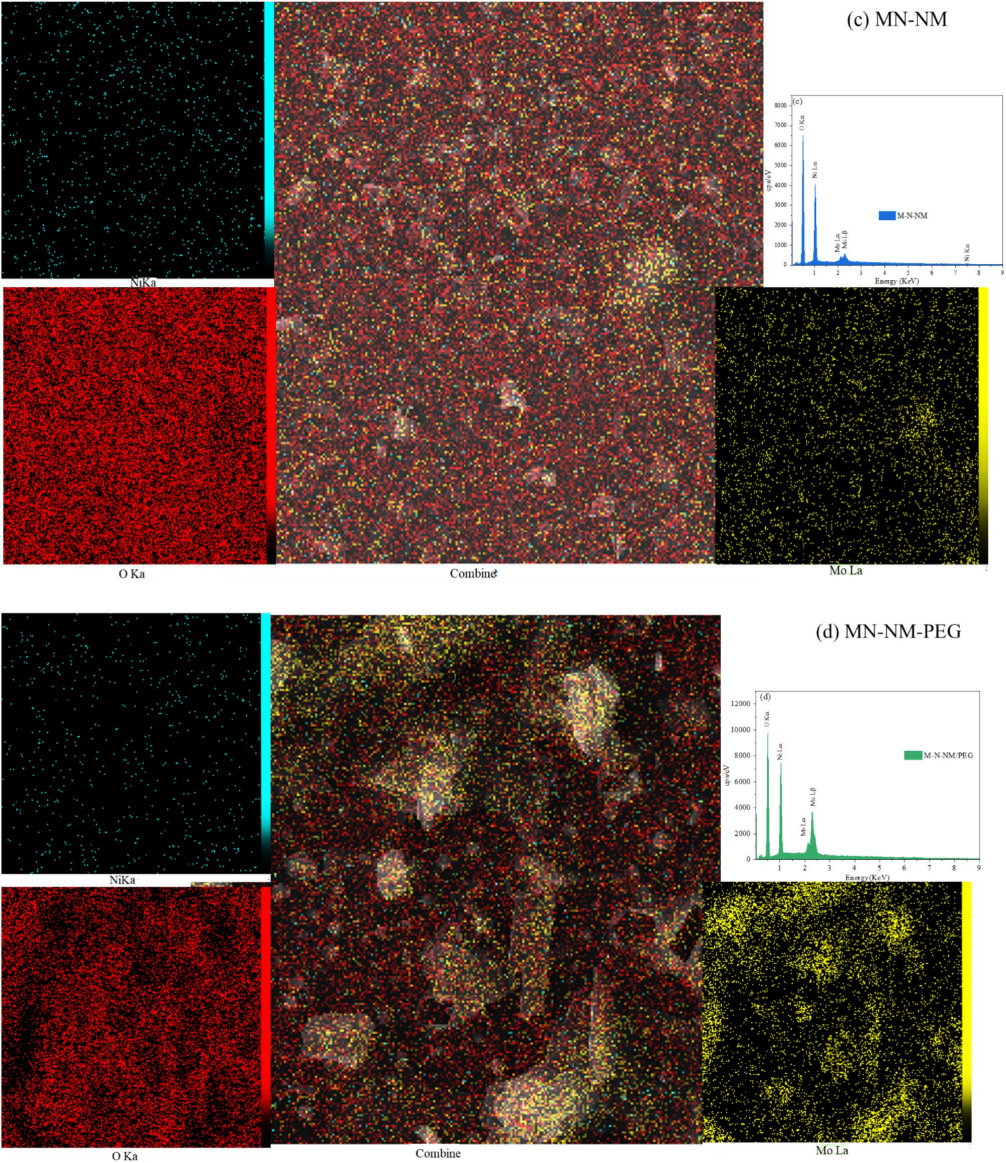

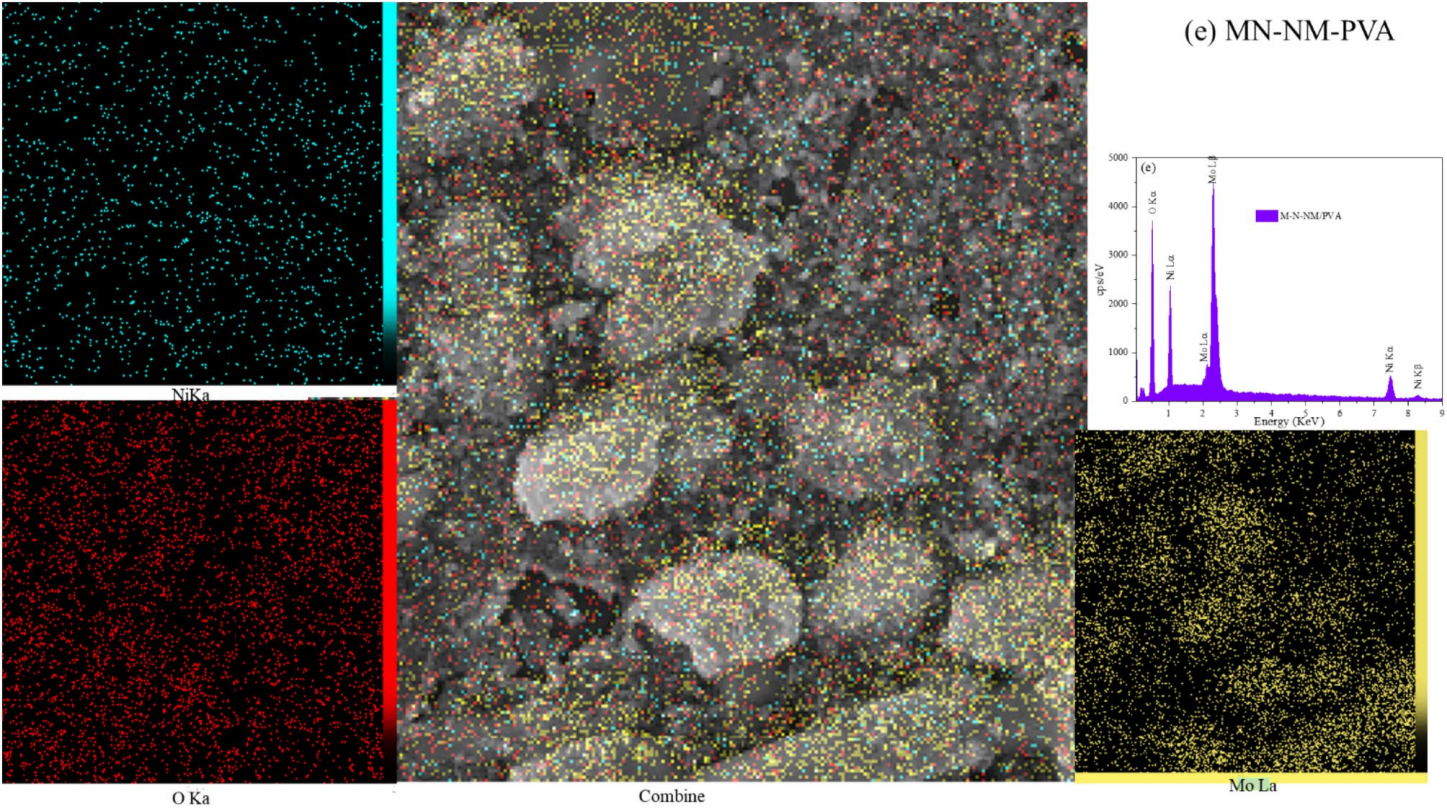
Distribution of Ni, Mo, and O in Samples using EDS mapping.

Figure 5 illustrates the elemental analysis of pure molybdenum trioxide (MoO_3_) and its composites with NiO, NiMoO_4_, and polymers. The composition of additives is known to impact composite materials’ physical and chemical properties, aiming to enhance their conductivity and capacitance. As illustrated in Fig. 5, the EDS mapping demonstrates the homogeneous distribution of Ni, Mo, and O across the selected area, providing. The mapping clearly confirms the presence of constituent elements such as nickel, molybdenum, and oxygen in the prepared composites.

### Morphological properties

#### Field emission scanning electron microscopy (FESEM)

**Fig. 6:**
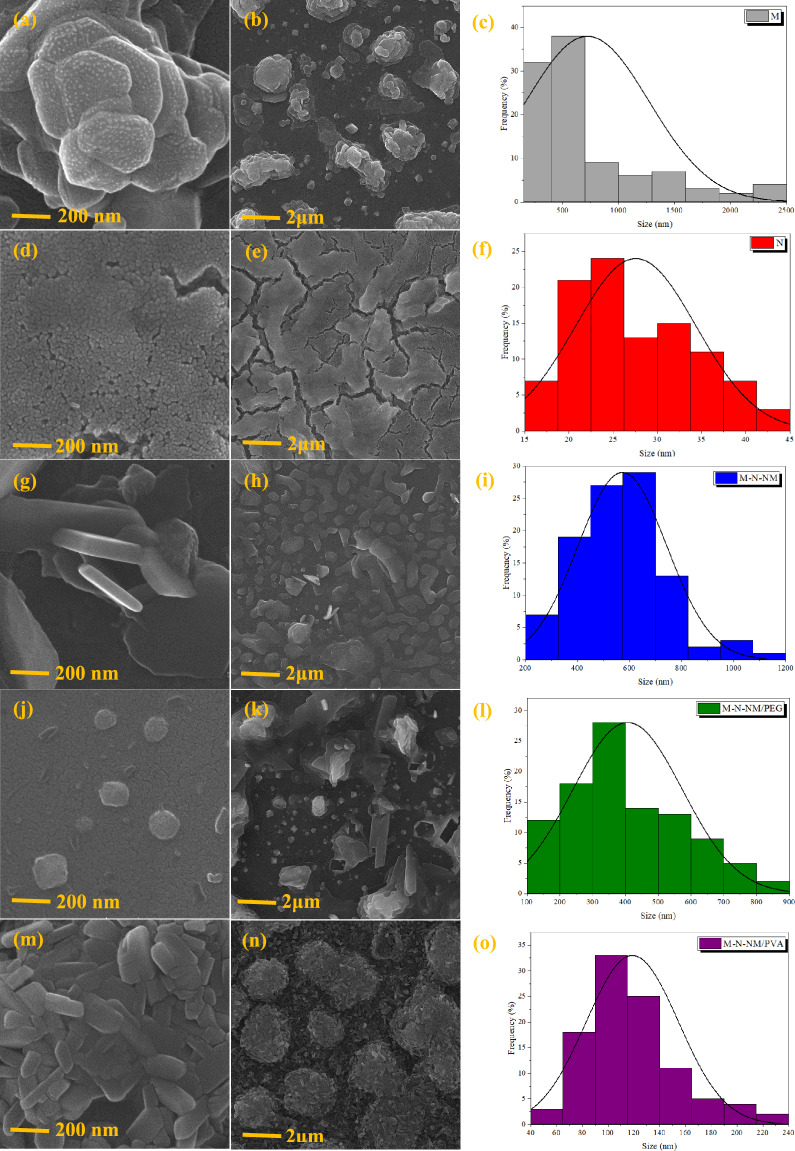
FESEM images of thin films deposited on the substrate at various magnifications and particle size distribution histograms (**a**, **b**, **c**) MoO_3_, (**d**, **e**, **f**) NiO, (**g**, **h**, **i**) MoO_3_-NiO-NiMoO_4_, (**j**, **k**, **l**) MoO_3_-NiO-NiMoO_4_/PEG, (**m**, **n**, **o**) MoO_3_-NiO-NiMoO_4_/PVA.

Figure 6 depicts the FESEM images of pure MoO_3_ and nanocomposites at various magnifications. The nanoporous morphology and small, uniformly distributed grains in the samples suggest an aggregated nanoparticle structure. The large agglomerate feature observed in the MoO_3_ films could be due to the high surface energy of MoO_3_ nanoparticles, leading to agglomeration during deposition^45^. The synthesized samples exhibit a surface abundant in mesopores and micropores. Also, The preparation conditions and choice of precursors significantly influence grains’ size and morphology of nanoparticles, so the incorporation of PEG results in creating a uniform surface. On the other hand, PVA gives a granular shape and a porous surface to the sample.

To gain a deeper understanding of the morphology and particle size of the synthesized nanocomposite thin films, SEM image analysis was performed, and the corresponding particle size distribution histograms are presented in Fig. 6. These histograms clearly demonstrate variations in average particle size and dispersion across the different samples. The incorporation of various additives, such as PEG and PVA, resulted in more uniform and smaller particle sizes, indicating their significant role in controlling nucleation and growth processes during film formation. This analysis further supports the morphological claims and highlights the effect of synthesis parameters on the microstructural properties of the films.

#### Atomic force microscopy (AFM)

**Fig. 7:**
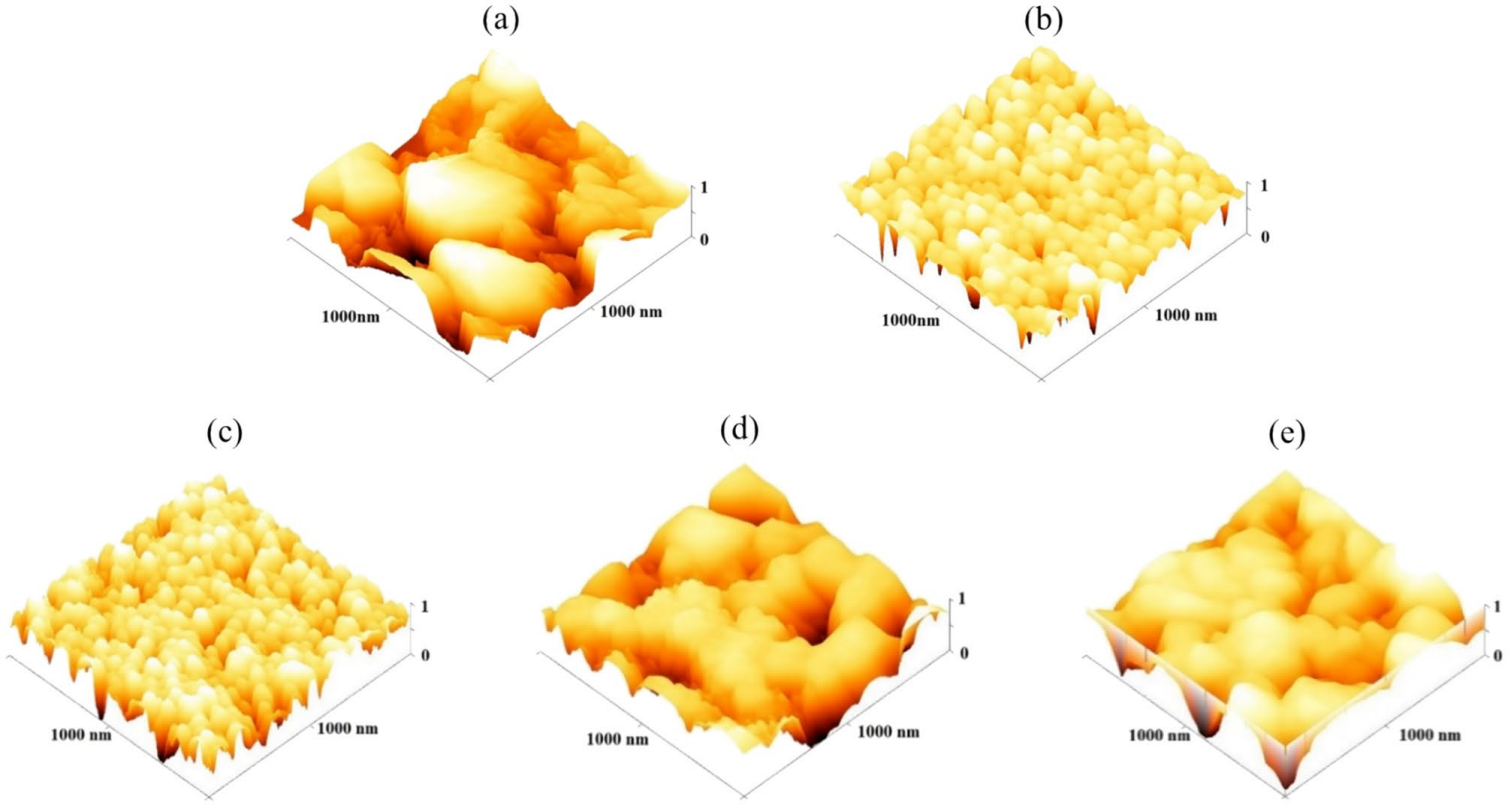
AFM images of (**a**) M, (**b**) N, (**c**) M-N-NM (**d**) M-N-NM/PEG (**e**) M-N-NM/PVA.

To describe the topography of the films, parameters such as the average maximum height of the profile (*R*_*z*_), root mean square roughness (*R*_*q*_), average surface roughness (*R*_*a*_), kurtosis (*R*_*ku*_), and Skewness (*R*_*sk*_) were calculated from the AFM images (Fig. 7). Based on the findings, the MoO_3_ thin film exhibits the highest surface roughness compared to all other samples, and an increase in surface roughness was observed upon the addition of the polymers. Conversely, NiO demonstrates the lowest roughness value.

The *R*_*sk*_ value indicates the symmetry of height dispensation around the average line, with a skewness value of zero indicating symmetrical height dispensation. It is observed that M-N-NM/PEG and M-N-NM/PVA exhibit negative *R*_*sk*_ values, suggesting a surface with valleys outnumbering peaks, thus indicating a predominance of valleys over peaks. Conversely, kurtosis assesses the trenchancy of the surface, with a value of roughness kurtosis (R_Ku_) of 3 representing Gaussian distribution. If R_Ku_ < 3, the surface is flat, while for values greater than 3, the surface is spiky^46^. The values of R_Ku_ exceed 3 for samples with polymers.

### Optical properties

**Fig. 8:**
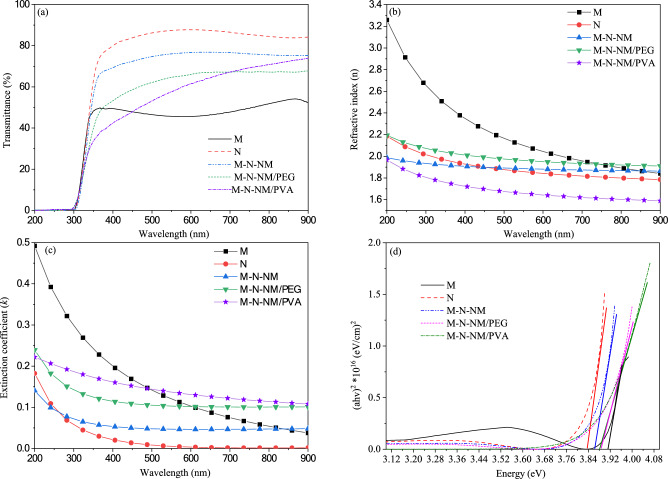
(**a**) The transmittance spectra of samples band, (**b**) Refractive index (*n*), (**c**) The extinction coefficient (*k*) of thin films, and (**d**) Variation in (*αhν*)^2^ against *hν* for all thin films.

Figure 8a displays the transmittance spectra of thin films across the wavelength range of 200–900 nm. Incorporating polymer resulted in a reduction in the transmittance of M-N-MN thin films. The alteration in thin film transmittance can be attributed to the surface roughness values detailed in Table 2. Augmenting the surface roughness induces greater light scattering, consequently diminishing the transmittance^47^. Due to the absence of interference fringes, the pointwise unconstrained minimization approach (PUMA) was used to calculate the refractive index and extinction coefficient^48^, as shown in Fig. 8b,c. The acquired data were fitted by the Cauchy relation tailored for samples. The findings reveal that the MoO_3_ thin film exhibits the highest extinction coefficient and refractive index. This happens because of the large size of MoO_3_ particles. The optical band gaps of the films were calculated by examining the transmittance spectra of the thin films. The band gap values were obtained through fundamental absorption, involving the excitation of electrons from the valence band to the conduction band. The absorption coefficient (*α*) is related to the optical band gap through the Tauc equation^49^:

**Table 2:** The statistical parameters of the specimens were computed for all samples.

Sample	*R*_z_(nm)	*R*_a_(nm)	*R*_q_(nm)	*R*_sk_	*R*_ku_
M	182.2	133	156	0.073	2.21
N	25.6	3.08	3.84	0.14	3.00
M–N-NM	99.3	10.9	13.9	0.065	2.61
M–N-NM/PEG	147.3	65.9	86.2	-0.10	3.32
M–N-NM/PVA	143.1	78.6	98.1	-0.073	3.95

6 $$\alpha h\nu =A{(h\nu -{E}_{\text{gT}})}^{n}$$

In Eq. 6, the photon energy is denoted by the symbol *hν*, *A* is a constant, and *n* represents the type of transition. The values of *n* can be 1/2, 2, 3, and 3/2, representing permitted direct, permitted indirect, prohibited indirect transitions, and prohibited direct, respectively. Using the following equations in the strong absorption region, *α* can be calculated^50^:

7 $$\alpha =-\frac{1}{d}\text{ln}x$$

8 $$x=\frac{{\left(n-1\right)}^{3}\left(n+{s}^{2}\right)T}{16{n}^{2}s}$$

Here, *T* represents the transmittance percentage, while *n* and *s* denote the film’s and substrate’s refractive index, respectively. The thickness of the films, *d*, is determined using the PUMA. The thickness values are 225 nm for M, 170 nm for N, 230 nm for M-N-NM, 335 nm for M-N-NM/PEG, and 350 nm for M-N-NM/PVA. Various parameters such as grain boundaries, structural defects, roughness, and thickness of layers can affect the absorption coefficient^51^.

From the extrapolation of the linear part of the plot of (*αhν*)^2^ versus *hν*, the direct band gap of the thin films was ascertained (Fig. 8d). The intersection point on the photon energy axis indicates the band gap. The resultant values are documented in Table 3. Employing the Tauc relation for direct band gap transitions, the optical band gap of the pure MoO_3_ film was estimated to be approximately 3.92 eV, as depicted in Fig. 8d. This determined band gap closely aligns with previously reported values in the existing literature on MoO_3_ material^52–54^. Also, our findings closely align with the band gap values reported for MoO_3_ and NiO fabricated via the sol–gel process^52,55,56^. The incorporation of polymer elevated the optical band gap of M-N-NM thin films, rendering them more responsive to wavelengths within the visible spectrum, thus enhancing their suitability for optical applications.

**Table 3:** Dielectric constants and dispersion parameters of thin films.

Sample	*E*_o_(eV)	*E*_d_(eV)	*M*_-1_	*M*_-3_(eV^−2^)	*ε*_∞_	*ε*_L_	*N*/m* × 10^38^ (Kg^−1^ m^−3^)	*E*_*u*_
M	5.52	14.16	2.57	0.084	3.57	4.58	12.94	0.06
N	7.49	16.12	2.15	0.038	3.15	3.40	0.961	0.12
M-N-NM	10.37	23.79	2.29	0.021	3.29	3.53	0.089	0.12
M-N-NM/PEG	8.70	22.41	2.58	0.034	3.58	3.77	0.206	0.16
M-N-NM/PVA	11.05	21.75	1.97	0.016	2.97	3.16	13.63	0.18

**Fig. 9:**
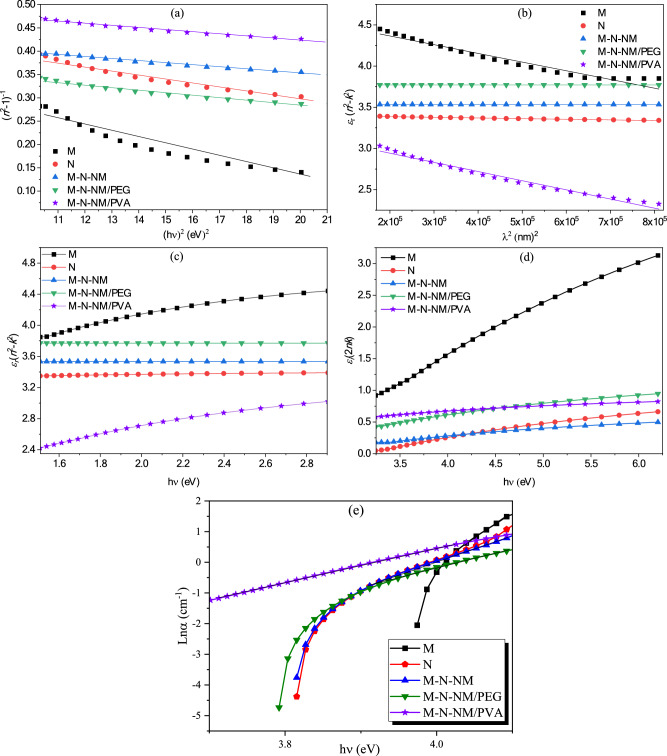
(**a**) plot of (*n*^2^-1)^-1^ versus photon energy squared (*hν*)^2^. (**b**) Plot of real dielectric constant (*ε*_r_) versus wavelength squared *λ*^2^, (**c**) spectral distribution of real part of the dielectric constant (*ε*_r_), (**d**) spectral distribution of imaginary part of the dielectric constant (*ε*_i_). (**e**) Urbach energy plots.

Using the single-oscillator model of Wemple-DiDomenico, the dispersion of the refractive index (*n*) can be evaluated^57^. The equation between the oscillator strength below the band gap and the refractive index (*n*) in the low absorption region is described as follows:

9 $${n}^{2}\left(h\nu \right)-1=\frac{{E}_{\text{o}}{E}_{\text{d}}}{{E}_{\text{o}}^{2}-{\left(h\nu \right)}^{2}}$$

where *E*_*0*_ represents the energy of the single oscillator due to the energy exchange between the valence and conduction bands, and *E*_*d*_ denotes scattering energy due to the average optical transmission power of the interband^58^. In the plot of *(n*^*2*^*-1)*^*-1*^ versus *(hν)*^*2*^ in Fig. 9a, parameters* E*_0_ and* E*_d_ are derived from the slope and the intersection of the linear part. By using* M*_-1_ and* M*_-3_ moments of the optical spectra, the inter-band transmission strengths can be evaluated, which are determined by the following equations^59,60^:

10 $${M}_{-1}=\frac{{E}_{d}}{{E}_{0}}$$

11 $${M}_{-3} =\frac{{E}_{d}}{{E}_{0}^{3}}$$

The obtained values of *E*_*0*_, *E*_*d*_, *M*_*-1*_, and *M-*_*3*_ for thin films are presented in Table 3. Additionally, the dielectric constant of infinite frequency *ε*_*∞*_ = *n*_*∞*_^*2*^ is obtained at *(hν)*^*2*^ = *0*^61^.

The complex dielectric constant, connects the electron transitions between bands in a solid to its structural characteristics and describes the optical response of the medium across all photon frequencies.

12 $${\varepsilon }^{*}\left(\nu \right)={\varepsilon }_{r}\left(\nu \right)+i{\varepsilon }_{i}\left(\nu \right)$$

Here, *ε*_*r*_ and *ε*_*i*_ indicate the dielectric constant real and imaginary parts, respectively. Their values are linked to the incident photon wavelength by the following relations^62^:

13 $${\varepsilon }_{r}\left(\nu \right)={n}^{2}-{k}^{2}$$

14 $${\varepsilon }_{i}\left(\nu \right)=2nk$$

To further analyze the optical properties, the susceptibility share of the free carrier electric (*χ*_*e*_) to the real dielectric constant (*ε*_*r*_) is examined using the model of Spitzer-Fan^63^:

15 $${\varepsilon }_{r}\left(\nu \right)={\varepsilon }_{L}-\left(\frac{{e}^{2}}{4{\pi }^{2}{c}^{2}{\varepsilon }_{0}}\right)\left(\frac{N}{{m}^{*}}\right){\lambda }^{2}$$

where *ε*_*L*_ is the dielectric constant of the lattice, (N/m*) is the ratio of carrier number to effective mass, *ε*_*0*_ is the vacuum permittivity, *c* is the light speed, and *e* is the electron charge. Figure 9b indicates the relationship between the real dielectric constant *ε*_*r*_ and *λ*^*2*^. The value of *ε*_*L*_ for samples is calculated by extrapolating the curves to the axis intercept, and the N/m* values are derived from the slope as per Eq. (15). The appraised values are prepared in Table 3. Figure 9c,d display the real part of the dielectric constant (*ε*_*r*_) and the imaginary part of the dielectric constant (*ε*_*i*_) as a function of incident photon energy (*hν*).

The Urbach energy (*E*_*u*_) is a key optical parameter that reflects the width of the exponential tail of the absorption edge in semiconducting materials. This tail originates from localized states within the bandgap caused by structural disorder, impurities, and defects. Evaluating *E*_*u*_ provides crucial insight into the defect density and degree of disorder in thin films, which significantly affects their optical and electronic properties, particularly in applications such as solar cells, LEDs, and sensors^64^. In this study, Urbach energy values were determined by plotting ln(*α*) versus photon energy (*hν*) and calculating the inverse slope of the linear region. As shown in Fig. 9e and summarized in Table 3, the M sample, with the lowest *E*_*u*_, possesses the highest structural order and the fewest localized defect states. On the other hand, the PEG and PVA samples show increased Urbach energies, indicating a higher degree of disorder likely caused by the introduction of amorphous polymeric content. The M-N-NM composite sample exhibits an intermediate value of 0.12 eV, suggesting that the synergistic interaction between MoO_3_, NiO, and NiMoO_4_ helps to moderate the disorder while preserving favorable optical properties. Overall, the Urbach energy analysis complements the bandgap study and provides a deeper understanding of how microstructural features influence the performance of these nanocomposite thin films.

**Fig. 10:**
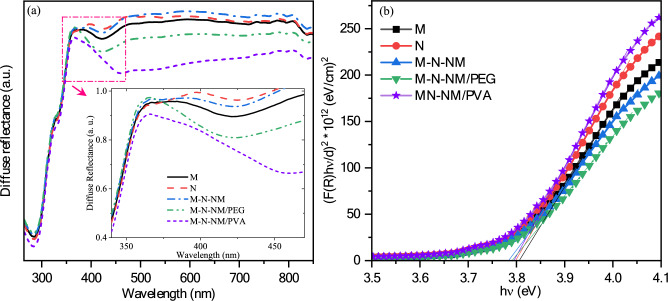
(**a**) diffuse reflectance spectra, (**b**) (F(*R*)*hν*/*d*)^2^ versus *hν*. for all thin films.

Figure 10 depicts the diffuse reflectance spectra of the samples prepared using the sol–gel spin-coating method. The diffuse reflection demonstrates a notable decline in the absorption edge, indicative of light transitions occurring within slot between distinct energy bands. Following the analysis of diffuse reflectance spectra (DRS), the Kubelka − Munk function was applied to determine the band gap energy precisely. That is expressed as follows^65^:

16 $$\text{F}\left(R\right)=\frac{{\left(1-R\right)}^{2}}{2R}=\frac{K}{S}$$

In Eq. 16, the diffuse reflectance is denoted by the symbol *R*, the effective absorption by *K*, and the scattering coefficient by *S*. And also, *F(R)* can be utilized to estimate the samples’ energy band gap (*E*_*gDRS*_). This estimation involves extrapolating the linear department of *(F(R)hν/d)*^*2*^ versus photon energy (*hν*), where *d* is the film thickness. The resulting energy band gap spectra are illustrated in Fig. 10b. Through this approach, *E*_*gDRS*_ is determined to be 3.82 eV for MoO_3_ film. Comparing the values in Table 4, there is minimal disparity between energy band gaps computed by diffuse reflectance spectroscopy (*E*_*gDRS*_) and those computed using Tauc plots from optical transmission spectra (*E*_*gT*_). Notably, band gaps calculated via diffuse reflectance spectroscopy are slightly smaller than those obtained through Tauc plot calculations. According to the EDX and XRD results, the pure orthorhombic phases are hypothesized to contain defects of grain boundary such as interstitial or vacancy of oxygen, leading to non-stoichiometric conduct^66^. Additionally, the decrease in reflectance intensity for M-N-NM/PVA is attributed to the effect of crystallite size on the scattering process. The enhanced size of crystallite substantially reduces the scattering coefficient and consequently diminishes the reflectance percent in the spectra^67^.

**Table 4:** The band gaps were calculated by diffuse reflectance spectroscopy (*E*_gDRS_) and optical transmission data (*E*_gT_).

Sample	*E*_gDRS_(eV)	*E*_gT_(eV)
M	3.82	3.92
N	3.80	3.84
M-N-NM	3.78	3.87
M-N-NM/PEG	3.79	3.89
M-N-NM/PVA	3.79	3.90

The structural role of polymer additives (PEG and PVA) in the MoO_3_–NiO–NiMoO_4_ nanocomposite matrix was clearly evidenced through multiple characterization techniques. The FESEM images revealed that the samples containing PEG and PVA exhibited finer surface morphology with enhanced porosity compared to the pristine nanocomposite.

XRD patterns showed a slight broadening of diffraction peaks in the PEG- and PVA-based samples, suggesting a reduction in crystallite size and an increase in structural disorder. Moreover, FTIR analysis indicated that after annealing, most of the polymer content was removed, but their templating effect remained, as evidenced by the increased surface roughness and porous texture. These structural modifications contribute to more accessible electroactive sites and facilitate ion transport during electrochemical processes, which is reflected in the improved CV, GCD, and EIS performance of the polymer-assisted electrodes. Therefore, PEG and PVA function not only as processing additives but also as key modifiers of the microstructure and functionality of the final electrodes.

### Electrochemical performance

**Fig. 11:**
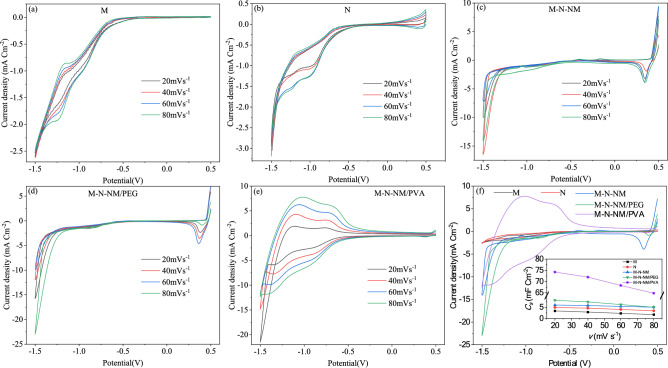
CV curves of (**a**) M, (**b**) N, (**c**) M-N-NM, (**d**) M-N-NM/PEG, (**e**) M-N-NM/PVA, (**f**) Comparison of CV curves of all electrodes at 80 mVs^− 1^. The inset shows the specific capacitance (*C*_s_) of the electrodes vs scan rate (*v*).

Cyclic voltammetry measurements were engaged to evaluate the electrochemical efficiency of the synthesized thin films. The Cyclic voltammetry was recorded for different scan rates (20, 40, 60, 80 mV s^−1^) within a potential window of 0.5 to −1.5 V at room temperature. Figure 11a–e illustrates normal CV curves for NiO, α-MoO_3_, and MoO_3_-NiO-NiMoO_4_ composites at different scan rates and Fig. 11f for all samples at a scan rate of 80 mV s^−1^. The symmetric CV curve, observed for all composite samples, signifies the ideal quasi-capacitive nature of the electrode material^68^. As depicted in Fig. 11a–c, there is an increase in both the integrated area and the specific capacitance (*C*_*s*_) beheld in the cyclic voltammogram of the M-N-NM electrode compared to the M and N electrodes. The M-N-NM composite electrode exhibits enhanced pseudocapacitive behavior, attributed to improved ionic and electronic conductivity through strong bonding between NiO and MoO_3_ nanoparticles. As a result, this facilitates a Faradaic process with higher efficiency of electrochemically active material on the electrodes. The consistent CV curve across all composite materials indicates positive synergistic effects and an enhanced pseudo-capacitive nature, particularly by adding PVA.

The peak currents show a gradual enhancement with increasing sweep rate. Even in a sweep rate of 80 mV s^−1^, the pair of redox peaks in the M-N-NM/PVA electrode is discernible, indicating superior reversibility cycle and rate performance compared to the other samples. The mechanism of charge storage of electrode materials is explored relying on the functional reaction. In N, N-M-NM, M–N-NM/PEG, and M–N-NM/PVA electrodes, the peaks identified within the 0–0.5 V potential range correspond to reactions of reversible redox involving Ni(III) ↔ Ni (II), while those within the − 1 to − 0.5 V potential range correspond to reactions of reversible redox of Ni ↔ Ni(II)^69^.

17 $$\text{NiO }+{\text{ OH}}^{-}\leftrightarrow \text{NiOOH}+{\text{e}}^{-}$$

18 $$\text{Ni}{(\text{OH})}_{2}+{\text{OH}}^{-}\leftrightarrow \text{NiOOH}+{\text{H}}_{2}\text{O}+{\text{e}}^{-}$$

As shown in Fig. 11, the CV curves of M, M-N-NM, M-N-NM/PEG, and M-N-NM/PVA exhibit a pair of redox peaks in − 1.5 to − 1, manifesting the Faradaic behavior of charge storage that can be related to the below reversible redox reactions^70^.

19 $${\text{MoO}}_{3}+x{\text{K}}^{+}+x{\text{e}}^{-}\leftrightarrow {\text{K}}_{x}{\text{MoO}}_{3}$$

The electrodes’ interfacial capacitance (*C*_*s*_) was determined from the CV pattern using Eq. (20). specific capacitance was calculated based on sample surface area (*A*) in the electrolyte, initial voltage (*V*_*i*_), final voltage (*V*_*f*_), current (*I*), and scan rate (*ν*)^71^.

20 $${C}_{s}\left({V}_{\text{f}}-{V}_{\text{i}}\right)={q}_{\text{S}}=\frac{1}{A\times \nu }{\int }_{{V}_{i}}^{{V}_{f}}I\left(\nu \right)d\nu$$

In all electrodes, the capacity declined as the scan rate increased, revealing a restriction in ion distribution at the active materials’ surface. Despite the intensification of redox peak intensities, their positions and shapes remained constant, indicating the electrode’s stability. The *C*_s_ of the electrodes vs scan rate (*v*) is shown in the inset of Fig. 11f.

An analytical method utilizing cyclic voltammetry was employed to gain a deeper understanding of the charge storage mechanism. By cycling these materials at varying sweep rates (20, 40, 60, 80 mV s^–1^), kinetic analysis enables the determination of the predominant charge storage mechanism, (whether surface-limited or diffusion-controlled). This relationship adheres to the Randless-Sevcik equation^72^:

21 $${I}_{\text{p}}=2.69\times {10}^{5}{n}^\frac{3}{2}S{D}^\frac{1}{2}C{\nu }^\frac{1}{2}$$

**Fig. 12:**
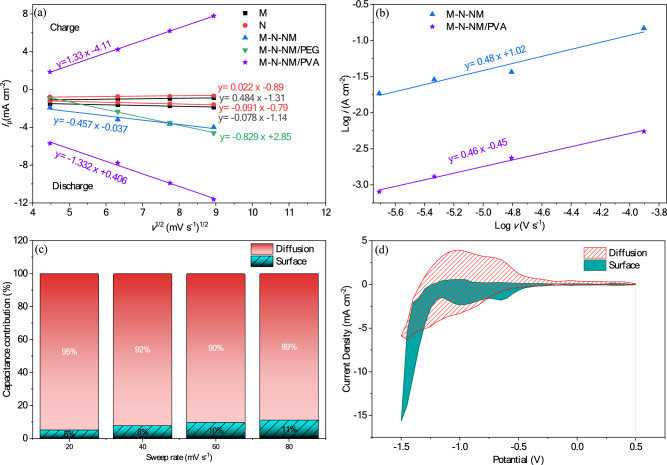
(**a**) The curves of the peak current versus the square root of scan rates, (b) The curves of log i versus log ν for reduction peaks of M-N-NM and M-N-NM/PVA, (c) The ratio of the diffusion-controlled mechanism and the surface capacitive effect at various sweep rates, and (d) the capacitive and diffusion contribution of CV curves at 20 mV s^−1^ for M-N-NM/PVA electrode.

Here, the peak redox current is denoted by the symbol *I*_*p*_, the initial concentration of OH‾ by *C*, the surface area of the electrode by *S*, the sweep rate by *v*, the diffusion coefficient by *D*, and the electron transfer number by *n*. The slope of the line observed in Fig. 12a is directly related to the diffusion coefficient (*D*). For M-N-NM/PVA, the oxidation reactions exhibit diffusion coefficients of approximately 4.5 times higher than the M-N-NM. As the scan rate increases, both the current response and the shift of redox peaks increase, suggesting possible control of the Faradaic reaction by ion diffusion. The storage mechanisms of the electrodes were explored by examining the relationship between scan rate (*ν*) and peak current (*i*) using Eqs. (22) and (23), with charge storage processes discernible through Dunn’s method and power-law relationship^73–75^.

22 $$i=a{\nu }^{b}$$

23 $$\text{log}(i) = \text{log}(a) + b \text{log}(\nu )$$

where, the symbol *i* represents current density, while *ν* denotes sweep rate, and *b* and *a* are tunable constants. Typically, from the slope of the plot of *log(i)* versus *log(ν)* at a given potential, the value of b can be determined. By analyzing the b-value extracted from plots of *log(i)* versus *log(ν)* at different redox potentials, the dominant charge-storage mechanism can be identified. A *b*-value close to 1 suggests a surface-controlled that indicates a pseudocapacitive behavior, whereas a value around 0.5 is characteristic of a battery-type material that indicates diffusion-controlled behavior. The linear correlation observed in Fig. 12b, with b-values around 0.5 for all electrodes, indicates that the electrochemical reactions are predominantly governed by ion diffusion processes, regardless of compositional variations^76^. Dunn’s equation is a suitable option to analyze the involvement of the diffusion-controlled workmanship and the surface capacitance effect at various sweep rates^75^:

24 $$i\left(\nu \right)={k}_{1}\nu +{k}_{2}{\nu }^\frac{1}{2}$$

where *k*_1_ and *k*_2_ are constants at a given potential, these constants are derived from a plot of *i*(*ν*)^-1/2^ versus *ν*^1/2^. This allows for the determination of the surface-limited (shaded region in Fig. 12c) and diffusion-limited contributions to the overall specific capacity.

**Fig. 13:**
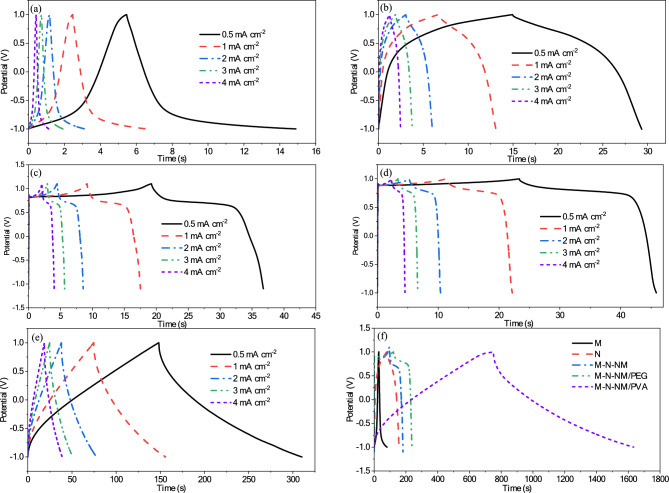
GCD curves of (**a**) M (**b**) N (**c**) M-N-NM (**d**) M-N-NM/PEG (**e**) M-N-NM/PVA electrode at different current densities (**f**) Comparison of GCD curves of all electrodes at a current density of 0.1 mA cm^−2^.

The capacitive and diffusion contribution of CV curves at 20 mV s^−1^ for M-N-NM/PVA electrode have been shown in Fig. 12d. The galvanostatic charge/discharge (GCD) curves of all samples are illustrated at the potential range from − 1 to 1 V for current densities spanning from 0.1 to 5 mA cm^−2^ (Fig. 13). The nonlinear shapes shown in these curves are related to Faradaic redox reactions that usually occur during the insertion/extraction of OH^-^ on the electrode surface and confirm the quasi-capacitive nature. Therefore, the GCD test is employed to determine the *C*_*s*_*,* (F cm^-2^), using Eq. 25 in the three-electrode system^77,78^.

25 $${C}_{s}=\frac{I\Delta t}{A\Delta V}$$

In the provided equations, *C*_s_ (F cm^-2^) represents specific capacitance, *I* stands for current (A), Δ*t* denotes total discharge time (s), *A* signifies the surface area of the electrode’s active materials (cm^2^), and Δ*V* indicates the potential window (V). Hence, it can be inferred that the compositions significantly influence the electrochemical response of the electrode reactions.

**Fig. 14:**
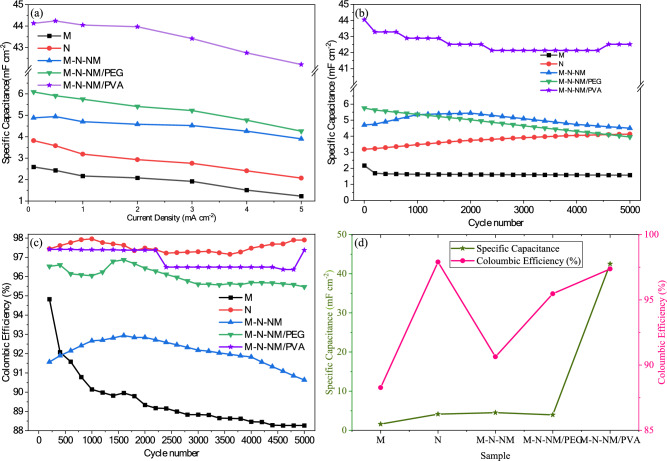
(**a**) Specific capacitance at various current densities (**b**) Cycling stability performance at a current density of 1 mA cm^2^. (**c**) Columbic efficiency plot of the three system electrodes. (**d**) Specific capacitance and Columbic efficiency plot of the three system electrodes after 5000 cycle number.

Figure 14a shows specific capacitance at various current densities. The cycling stability performance at a current density of 1 mA cm^2^ is given in Fig. 14b. Figure 14c illustrates the coulombic efficiency (CE) of the samples plotted against the cycle number. CE is defined as the ratio of the ions moving from one electrode to the other during charge to those returning during discharge, and it is estimated using the equation provided below^78^:

26 $$\eta =\frac{{\tau }_{d}}{{\tau }_{\text{c}}}\times 100 \%$$

In this equation, the coulombic efficiency is denoted by the symbol *η*, the charge time by *t*_*d*_, and the discharge time by *t*_*c*_. In each charge/discharge cycle, high coulombic efficiency indicates less capacity loss^79^.

Figure 14d shows specific capacitance and Columbic efficiency plots of the three system electrodes after 5000 cycle numbers. The cycling durability of the three electrodes was assessed at 5 mA cm^−2^ following 5000 cycles. As depicted in Fig. 14, the M-N-NM/PVA electrode demonstrates significantly superior capacitance retention (97.3%) compared to the other electrodes. This enhancement can be attributed to the presence of PVA, which acts as a structure-directing agent during film formation, resulting in a more interconnected and porous architecture. Such morphology facilitates efficient ion transport and electron conduction while maintaining structural stability during repeated cycling. The reduced internal resistance and improved electrolyte accessibility contribute to minimizing energy losses, thereby improving the overall Coulombic efficiency^80^.

To provide a clearer and more comprehensive comparison of electrochemical performance, the specific capacitance values obtained from both cyclic voltammetry (CV) and galvanostatic charge–discharge (GCD) measurements have been tabulated and presented in Table 5. The inclusion of this table allows for a direct quantitative comparison between the two methods. This dual representation offers better insight into the charge storage capability and consistency of the electrode materials under different testing conditions.

**Table 5:** Comparison of specific capacitance values obtained from CV and GCD techniques for different samples.

Sample	CV specific capacitance (mF cm^−2^) @20 mVs^−1^	GCD specific capacitance (mF cm^−2^) @0.1 mAcm^−2^
M	3.39	2.59
N	4.91	3.82
M-N-NM	6.05	4.88
M-N-NM/PEG	8.14	6.09
M-N-NM/PVA	74.22	44.13

**Fig. 15:**
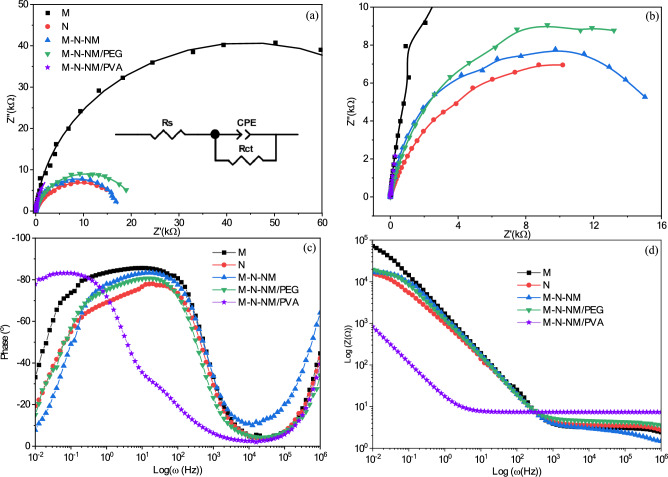
(**a**) The whole frequency and (**b**) the high frequency EIS Nyquist plots. (**c**) The Bode magnitude and (**d**) phase plots of electrodes. The equivalent circuits are shown in the inset.

Electrochemical impedance spectroscopy (EIS) emerges as a prominent technique for assessing electrode behavior, offering insights into the dynamics of charge movement across the electrode–electrolyte interface and conductance. EIS test was employed to evaluate the frequency-dependent behavior within the range of 0.01 Hz to 1 MHz. In electrochemical investigations, the utilization of Nyquist plots for assessing system stability is deemed appropriate. The Nyquist plots illustrating electrode characteristics are depicted in Fig. 15a,b. Based on these plots, a physically meaningful circuit model was defined, aiming to streamline the number of variables involved. This model, inclusive of elements such as charge-transfer resistance (*R*_*ct*_), equivalent series resistance (*R*_*s*_), and constant phase element (*CPE*), was utilized for fitting the Nyquist plots. The fitting process was carried out employing Zview software, resulting in the derivation of parameters (*R*_*ct*_*, R*_*s*_*, CPE*) which were documented in Table 6.

**Table 6:** Values for impedance parameters of electrodes in KOH electrolyte.

Sample	*R*_s_(Ω)	CPE-T(mF cm^-2^)	CPE-P	*R*_ct_(kΩ cm^-2^)
M	30.33	0.12	0.946	88.7
N	34.21	0.21	0.805	18.7
M–N-NM	30.14	0.14	0.925	17.5
M–N-NM/PEG	44.72	0.17	0.892	21.3
M–N-NM/PVA	73.76	2.92	0.961	35.4

Typically, the components employed in the equivalent electrical circuit carry these interpretations: *R*_s_ represents the point of intersection with the z’ axis denoting the resistance at the electrode–electrolyte interface, encompassing intrinsic electrode resistance, bulk and ionic resistance of the electrolyte, and current collector contact resistance^79^. The constant phase element is outlined in the formula (27)^81,82^:

27 $${Z}_{CPE}=\frac{1}{Q{\left(j\omega \right)}^{n}}$$

where *Q* is the pseudocapacitance, *ω(*= *2πf*) is the angular frequency, *j* is equal to *√ − 1,* and *n* is an empirical constant The parameters *Q* and *n* have already been introduced as CPE-T and CPE-P, respectively. The characterization of constant phase element (CPE) components depends upon the value of *n*. If *n* equals zero, the CPE behaves entirely resistively. Conversely, when *n* equals one, the CPE exhibits capacitive behavior, demonstrating an intermediate response between these two extremes when *0* < *n* < *1*. From Eq. (28), the actual capacity can be calculated^83^:

28 $$C={R}^{(\frac{1-n}{n})}{Q}^\frac{1}{n}$$

CPE, representing double-layer capacitance, is defined by two parameters: CPE-T and CPE-P. When CPE-P equals one, the formula mirrors that of a capacitor (*C*). Alternatively, when CPE-P is equal to 0.5, it generates a 45-degree line on the graph of the complex plane. Placing a CPE in parallel is depicted as a depressed semicircle, such as *R*_ct_, indicating charge transfer resistance across high and medium frequency regions. The diffusion coefficient, particle concentration, and kinetic effects are among the physical and chemical factors of the metal-electrolyte interface or related system, which can be attributed to the circuit formulated for all three electrodes. Figure 15c,d show the Bode magnitude and phase plots of the films, respectively. The Bode magnitude and phase plots of all the samples in the electrolyte showed similar trends with an exception for M-N-NM/PVA sample.

### Wettability

**Fig. 16:**
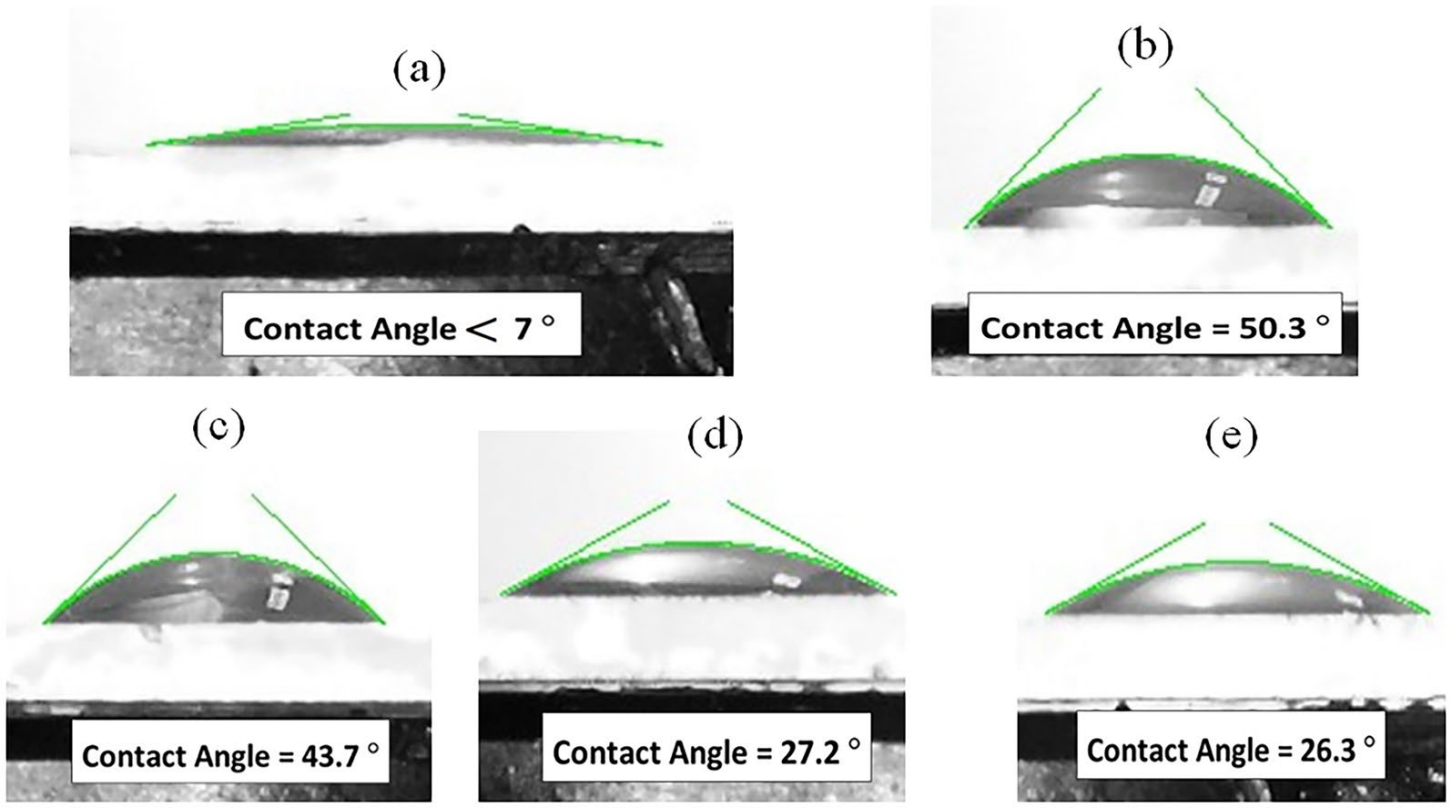
The wetting behavior of (**a**) M, (**b**) N, (**c**) M-N-NM (**d**) M-N-NM/PEG (**e**) M-N-NM/PVA.

To explore the wetting characteristics of the prepared films, we conducted contact angle measurements with water droplets on their surfaces, as depicted in Fig. 16. The contact angles obtained for M, N, M-N-NM, M-N-NM/PEG, and M-N-NM/PVA thin films are less than 7°, 50.3°, 43.7°, 27.2°, and 26.2°, respectively. Following the convention that 10 < θ < 90 indicates hydrophilicity, 90 < θ < 120 indicates hydrophobicity, θ < 10 indicates super hydrophilicity, and θ > 120 indicates super hydrophobicity^84^. Our findings indicate the hydrophilic nature of the samples. Moreover, the data reveal that the MoO_3_ film exhibits superhydrophilic properties. Superhydrophilic surfaces are valuable for their self-cleaning^85–88^, anti-fog^89–92^, anti-reflective^93–95^, and electrochemical behavior.

Several theoretical frameworks have emerged to clarify the wettability dynamics of surfaces. In the model presented by Wenzel, he believed that the liquid can completely penetrate the pores of the surface and make contact with the concave features of the surface. Wenzel amended Young’s formula by introducing a roughness factor, resulting in the modified formula^96^:

29 $$\text{Cos}{\theta }_{\text{W}}=r \text{Cos}\theta$$

where *θ*_*W*_ represents the apparent contact angle of a nonuniform surface, and *r* is a factor of the ratio of the actual area to the predicted area. According to Wenzel’s model, increasing the roughness of a hydrophilic surface enhances its hydrophilicity. In scenarios where air becomes trapped by the liquid, forming a composite surface, it falls under Cassie’s case and described the following modified formula for the apparent contact angle^97^.

30 $$\text{Cos}{\theta }_{\text{W}}={f}_{1} \text{Cos}\theta -{f}_{2}$$

Here, *f*_*1*_ represents the fraction of solid surface areas, and *f*_*2*_ represents the fraction of air trapped between the solid surface and the water drop, which is *f*_1_ + *f*_2_ = 1. It is clear from this model that the larger the air fraction (*f*_2_), the surface is more hydrophobic. According to the *R*_*a*_ values obtained from AFM analysis and the results of wettability, roughness and contact angle are completely related and the wetting behavior of the samples follows Wenzel’s model.

## Conclusion

The synthesis of MoO_3_-NiO-NiMoO_4_ nanocomposites on glass and FTO substrates using the sol–gel spin coating technique for energy storage applications was successfully achieved. Various characterization techniques including XRD, FTIR, and EDX were employed to comprehensively understand the microstructure, chemical composition, and morphology of the synthesized materials. XRD analysis revealed that MoO_3_-NiO-NiMoO_4_ composites exhibit a three-phase nature with distinct α-MoO_3_, NiO, and NiMoO_4_ phases. The average crystallite sizes ranged from 39.7 nm (N) to 58.2 nm (M-N-NM/PVA), and strain values varied between 0.13 and 0.28. Among the MoO_3_-NiO-NiMoO_4_ composites, the M-N-NM/PVA sample exhibits the highest roughness (58.2 nm) and the lowest contact angle (26.2°), indicating the best wettability. The transmittance spectra show that adding polymers reduces transmittance due to increased surface roughness. MoO_3_ films exhibit the highest refractive index and extinction coefficient due to their large particle size. The polymers also decrease the optical band gap, making the films more suitable for optical applications. Using a three-electrode cell configuration and through analyses like electrochemical impedance spectroscopy, cyclic voltammetry, and galvanostatic discharge techniques, the electrochemical behavior of the α-MoO_3_, NiO, and NiMoO_4_ composite materials was extensively investigated. Enhanced ionic conductivity was observed in all composite materials, leading to improved electrochemical performance and more ionic transfer at the electrode–electrolyte junction, as observed by EIS. Moreover, the composites electrode stability during long cycling exhibited significant enhancement compared to pristine MoO_3_ and NiO electrodes. The results demonstrate that the prepared M-N-NM/PVA electrode exhibits a maximum specific capacitance of 44.13 mF cm^-2^ at 0.1 mA cm^-2^, with a coulombic efficiency of 97.36% after 5000 cycles. The incorporation of PEG and PVA significantly enhanced the structural and electrochemical performance of the films, with PVA-based samples showing the highest capacitance. These findings highlight the role of polymer additives in optimizing thin-film electrodes for energy storage applications.

## Data Availability

The datasets used and/or analysed during the current study available from the corresponding author on reasonable request.

## References

[1] Strielkowski, W., Civín, L., Tarkhanova, E., Tvaronavičienė, M. & Petrenko, Y. Renewable energy in the sustainable development of electrical power sector: A review. *Energies* **14**, 8420. 10.3390/en14248240 (2021).

[2] Biswas, D. et al. Positron annihilation and correlated dielectric property studies of a transition metal oxide-modified quaternary nanocomposite 0.1P2O5–0.4ZnO–0.5(xV2O5–(1–x)MoO3). *J. Alloy Compd.* **864**, 158395. 10.1016/j.jallcom.2020.158395 (2021).

[3] Aghaei, F., Ghodsi, F. E. & Mazloom, J. Fabrication and investigation of structural, morphological properties, and electrochemical performance of MoO_3_-Bi_2_O_3_-Bi_2_MoO_6_ nanocomposites. *Ceram. Int.* **02**, 26. 10.1016/j.ceramint.2025.02.062 (2025).

[4] An, N. et al. Rational electrochemical design of cuprous oxide hierarchical microarchitectures and their derivatives for SERS sensing applications. *Small Methods* **8**, 2300910. 10.1002/smtd.202300910 (2024). 10.1002/smtd.202300910 38415973

[5] Lu, L., Le, J., Zhang, L., Shen, S. & Ren, X. Polarization independent tunable near-perfect absorber based on graphene-BaO arrays and Ag-dielectric Bragg reflector composite structure. *Diam. Relat. Mater.* **152**, 111958. 10.1016/j.diamond.2025.111958 (2025).

[6] Li, X., Liu, Y. & Leng, J. Large-scale fabrication of superhydrophobic shape memory composite films for efficient anti-icing and de-icing. *Sustain. Mater. Technol.* **37**, e00692. 10.1016/j.susmat.2023.e00692 (2023).

[7] Paz de Araujo, C. A. et al. Universal non-polar switching in carbon-doped transition metal oxides (TMOs) and post TMOs. *APL Mater.* **10**, 040904. 10.1063/5.0073513 (2022).

[8] Delbari, S. A. et al. Transition metal oxide-based electrode materials for flexible supercapacitors: A review. *J. Alloy Compd.* **857**, 158281. 10.1016/j.jallcom.2020.158281 (2021).

[9] Okpara, E. C., Olatunde, O. C., Wojuola, O. B. & Onwudiwe, D. C. Applications of transition metal oxides and chalcogenides and their composites in water treatment: a review. *Environ. Adv.* **11**, 100341. 10.1016/j.envadv.2023.100341 (2023).

[10] Yıldırım, F., Rouhi, H. F., Chenari, H. M., Biber, M. & Aydoğan, Ş. Robust electrospun Si@PVA-MoS₂:NiO nanofibers heterojunction for enhanced self-powered broadband photodetection. *Sens. Actuat. Phys.*10.1016/j.sna.2025.116616 (2025).

[11] Zheng, G. et al. Construction of three-dimensional crumpled Ni-Co TMOs for electrochemical energy storage. *Electroanalysis* **35**, e202200480. 10.1002/elan.202200480 (2023).

[12] Shaheen, I. et al. Recent advancements in metal oxides for energy storage materials: Design, classification, and electrodes configuration of supercapacitor. *J. Energy Storage* **72**, 108719. 10.1016/j.est.2023.108719 (2023).

[13] Li, W., Shi, J., Zhang, K. H. & MacManus-Driscoll, J. L. Defects in complex oxide thin films for electronics and energy applications: challenges and opportunities. *Mater. Horiz.* **7**, 2832. 10.1039/D0MH00899K (2020).

[14] Shao, M. et al. High-performance biodegradable energy storage devices enabled by heterostructured MoO_3_–MoS_2_. *Compos. Small* **19**, 2205529. 10.1002/smll.202205529 (2023). 10.1002/smll.202205529 36508711

[15] Avani, A. & Anila, E. Recent advances of MoO_3_ based materials in energy catalysis: Applications in hydrogen evolution and oxygen evolution reactions. *Int. J. Hydrogen Energy* **47**, 20475. 10.1016/j.ijhydene.2022.04.252 (2022).

[16] Cai, P. et al. An ultraviolet-deposited MoO_3_ film as anode interlayer for high-performance polymer solar cells. *Adv. Mater. Interfaces* **7**, 1901912. 10.1002/admi.201901912 (2020).

[17] Han, Q., Wang, R., Zhu, H., Wan, M. & Mai, Y. The preparation and investigation of all thin film electrochromic devices based on reactively sputtered MoO_3_ thin films. *Mat. Sci. Semicon. Proc.* **126**, 105686. 10.1016/j.mssp.2021.105686 (2021).

[18] Sharma, R., Sharma, A. K., Jha, R. & Sarkar, A. Stable α-MoO_3_ nanocrystals and its doped variants with unique morphologies under optimized reaction conditions for efficient electrochemical and photochromic performances. *Mater. Chem. Phys.* **280**, 125813. 10.1016/j.matchemphys.2022.125813 (2022).

[19] Sreelakshmi, V., Kaliani, A. A. & Jithin, M. Study of thermochromic and photocatalytic properties of MoO_3_ thin films. *Superlattice Microst.* **161**, 107096. 10.1016/j.spmi.2021.107096 (2022).

[20] Fan, Z., Zhang, X., Zhou, M., Yang, Y. & Wen, G. High sensitivity ethanol sensor based on MoO_3_ nanoparticles and its sensing mechanism. *J. Mater. Sci. Mater. El.* **34**, 275. 10.1007/s10854-022-09696-3 (2023).

[21] Sun, J., Meng, Y. & Zhang, B. Tribological behaviors and lubrication mechanism of water-based MoO_3_ nanofluid during cold rolling process. *J. Manuf. Process.* **61**, 518. 10.1016/j.jmapro.2020.11.044 (2021).

[22] Shi, M. et al. Temperature-controlled crystal size of wide band gap nickel oxide and its application in electrochromism. *J. Peng Micromach.* **12**, 80. 10.3390/mi12010080 (2021). 10.3390/mi12010080 PMC7828803 33466688

[23] Adiba, A., Pandey, V., Munjal, S. & Ahmad, T. Structural and optical properties of sol gel synthesized NiO nanoparticles. *AIP Conf. Proc. AIP Pub.* **2270**, 110011. 10.1063/5.0020038 (2020).

[24] Godlaveeti, S. K., El-marghany, A., Nagireddy, R. R., Gedi, S. & Chintaparty, R. Comparative study of electrochemical supercapacitor performance Among various nickel phases: Hydroxide, oxide, and sulfide. *Ceram. Int.* **02**, 229. 10.1016/j.ceramint.2025.02.229 (2025).

[25] Zhang, Y. et al. Influence of metallic oxide on the morphology and enhanced supercapacitive performance of NiMoO_4_ electrode material. *Inorg. Chem. Commun.* **112**, 107697. 10.1016/j.inoche.2019.107697 (2020).

[26] Truc, N. T. T. et al. Advanced NiMoO_4_/g-C_3_N_4_ Z-scheme heterojunction photocatalyst for efficient conversion of CO_2_ to valuable products. *J. Alloy Compd.* **842**, 155860. 10.1016/j.jallcom.2020.155860 (2020).

[27] Rammal, M. B. & Omanovic, S. Synthesis and characterization of NiO, MoO_3_, and NiMoO_4_ nanostructures through a green, facile method and their potential use as electrocatalysts for water splitting. *Mater. Chem. Phys.* **255**, 123570. 10.1016/j.matchemphys.2020.123570 (2020).

[28] Qian, F. et al. An overview of polylactic acid (PLA) nanocomposites for sensors. *Adv. Compos. Hybrid Mater.* **7**(3), 75. 10.1007/s42114-024-00887-6 (2024).

[29] Khanna, D. et al. Microwave processed metal nanoparticles/MoO_3_-PVA photoactive nano-composites for energy applications. *Mater. Sci. Eng.* **297**, 116822. 10.1016/j.mseb.2023.116822 (2023).

[30] Kundu, M. et al. A rational preparation strategy of phase tuned MoO_3_ nanostructures for high-performance all-solid asymmetric supercapacitor. *J. Energy Chem.* **87**, 192. 10.1016/j.jechem.2023.08.014 (2023).

[31] Nath, D., Singh, F. & Das, R. X-ray diffraction analysis by Williamson-Hall, Halder-Wagner and size-strain plot methods of CdSe nanoparticles- a comparative study. *Mater. Chem. Phys.* **239**, 122021. 10.1016/j.matchemphys.2019.122021 (2020).

[32] Singh, J., Mimani, T., Patil, K. C. & Bhat, S. V. Enhanced lithium-ion transport in PEG-based composite polymer electrolyte with Mn_0.03_Zn_0.97_Al_2_O_4_ nanoparticles. *Solid State Ion* **154**, 21–27. 10.1016/S0167-2738(02)00698-7 (2002).

[33] Rouhi, H. F. & Chenari, H. M. Two-dimensional (2D) MoS_2_-nanosheet (NS) incorporated within electrospun PVA nanofibers: Fabrication and characterization study. *J. Surf. Interface* **51**, 104702. 10.1016/j.surfin.2024.104702 (2024).

[34] Chibane, L., Belkaid, M. S., Zirmi, R. & Moussi, A. A simple process for synthesis of transparent thin films of molybdenum trioxide in the orthorhombic phase (α-MoO_3_). *J. Electron Mater.* **46**, 1963. 10.1007/s11664-016-5239-1 (2017).

[35] Balaji, M., Chandrasekaran, J. & Raja, M. Role of substrate temperature on MoO3 thin films by the JNS pyrolysis technique for P-N junction diode application. *Mat. Sci. Semicon. Proc.* **43**, 104. 10.1016/j.mssp.2015.12.009 (2016).

[36] Alharbi, E. M. & Rajeh, A. Tailoring the structural, optical, dielectric, and electrical properties of PEO/PVA blend using graphene nanoplates for energy storage devices. *J. Mater. Sci. Mater. Electron* **28**, 22196–22207. 10.1007/s10854-022-08999-9 (2022).

[37] Xie, R., Huang, H., Qi, X. & Wei, G. Significant enhancement of the electrochemical performance of hierarchical Co_3_O_4_ electrodes for supercapacitors via architecture design and training activation. *J. Energy Storage* **35**, 102258. 10.1016/j.est.2021.102258 (2021).

[38] Wongkrua, P., Thongtem, T. & Thongtem, S. Synthesis of h- and α-MoO_3_ by refluxing and calcination combination: Phase and morphology transformation, photocatalysis, and photosensitization. *J. Nanomater.* **2013**, 702679. 10.1155/2013/702679 (2013).

[39] Song, Y., Zhao, Y., Huang, Z. & Zhao, J. Aqueous synthesis of molybdenum trioxide (h-MoO_3_, α-MoO_3_·H_2_O and h-/α-MoO_3_ composites) and their photochromic properties study. *J. Alloy. Compd.* **693**, 1290. 10.1016/j.jallcom.2016.10.092 (2017).

[40] Hu, H., Deng, C., Xu, J., Zhang, K. & Sun, M. Metastable h-MoO3 and stable α-MoO_3_ microstructures: controllable synthesis, growth mechanism and their enhanced photocatalytic activity. *J. Exp. Nanosci.* **10**, 1336. 10.1080/17458080.2015.1012654 (2015).

[41] Drozdowska, K., Welearegay, T., Österlund, L. & Smulko, J. High performance acetone gas sensor based on ultrathin porous NiO nanosheet. *Actuat. B Chem.* **353**, 131125. 10.1016/j.snb.2021.131125 (2022).

[42] Khodair, Z. T., Noor, M., Ibrahim, T., Kadhim, J. & Mohammad, A. M. Synthesis and characterization of nickel oxide (NiO) nanoparticles using an environmentally friendly method, and their biomedical applications. *Chem. Phys. Lett.* **797**, 139564. 10.1016/j.cplett.2022.139564 (2022).

[43] Popovych, O. et al. Methods of obtaining nickel molybdates and composites of molybdate/carbon material for electrodes of hybrid supercapacitors (Review). *Phys. Chem. Solid* **21**, 650. 10.15330/pcss.21.4.650-659 (2020).

[44] Ramulu, B., Sekhar, S. C., Nagaraju, G. & Yu, J. S. Rational design and construction of nickel molybdate nanohybrid composite for high-performance supercapattery. *Appl. Surf. Sci.* **515**, 146023. 10.1016/j.apsusc.2020.146023 (2020).

[45] Aqaei, F., Zare, M. & Shafiekhani, A. Role of plasmonic Au nanoparticles embedded in the diamond-like carbon overlayer in the performance of CuFeO_2_ solar photocathodes. *J. Solid State Electrochem.* **25**, 1139. 10.1007/s10008-020-04876-9 (2021).

[46] Aguilar-Morales, A. I., Alamri, S., Kunze, T. & Lasagni, A. F. Influence of processing parameters on surface texture homogeneity using direct laser interference patterning. *Opt. Laser Technol.* **107**, 216. 10.1016/j.optlastec.2018.05.044 (2018).

[47] Bazhan, Z., Ghodsi, F. E. & Mazloom, J. Modified voltammetric, impedimetric and optical behavior of polymer- assisted sol-gel MgFe_2_O_4_ nanostructured thin films. *Electrochim. Acta* **250**, 143. 10.1016/j.electacta.2017.08.026 (2017).

[48] Zubair, M. & Chowdhury, M. A dynamic optical constant extraction method for thin films with structural and optical-parametric justifications. *J. Appl. Phys.* **128**, 195301. 10.1063/5.0027370 (2020).

[49] Mergen, Ö. B. & Arda, E. Determination of optical band gap energies of CS/MWCNT Bio-nanocomposites by Tauc and ASF methods. *Syn. Met.* **269**, 116539. 10.1016/j.synthmet.2020.116539 (2020).

[50] Bouzidi, A., Omri, K., Jilani, W., Guermazi, H. & Yahia, I. S. Influence of TiO_2_ incorporation on the microstructure, optical, and dielectric properties of TiO_2_/Epoxy composites. *J. Inorg. Organomet. Polym. Mater.* **28**, 1114. 10.1007/s10904-017-0772-9 (2018).

[51] Ciambriello, L., Cavaliere, E. & Gavioli, L. Influence of roughness, porosity and grain morphology on the optical properties of ultrathin Ag films. *Appl. Surf. Sci.* **576**, 151885. 10.1016/j.apsusc.2021.151885 (2022).

[52] Layegh, M., Ghodsi, F. E. & Hadipour, H. Improving the electrochemical response of nanostructured MoO_3_ electrodes by Co doping: Synthesis and characterization. *J. Phys. Chem. Solid.* **121**, 375. 10.1016/j.jpcs.2018.05.044 (2018).

[53] Egbo, K. O. et al. Band alignment of wide bandgap NiO/MoO_3_ and NiO/WO_3_ p-n heterojunctions studied by high-resolution X-ray photoelectron spectroscopy. *J. Alloy. Compd.* **876**, 160136. 10.1016/j.jallcom.2021.160136 (2021).

[54] Pavoni, E. et al. First-principles calculation of MoO_2_ and MoO_3_ electronic and optical properties compared with experimental data. *Nanomaterials* **13**, 1319. 10.3390/nano13081319 (2023). 37110904 10.3390/nano13081319 PMC10144520

[55] Bonomo, M. Synthesis and characterization of NiO nanostructures: a review. *J. Nanopar. Res.* **20**, 222. 10.1007/s11051-018-4327-y (2018).

[56] Salim, E. & Tarabiah, A. E. The influence of nio nanoparticles on structural, optical and dielectric properties of CMC/PVA/PEDOT:PSS nanocomposites. *J. Inorg. Organomet. Polym. Mater.* **6**, 1638. 10.1007/s10904-023-02591-2 (2023).

[57] Abd-Elnaiem, A. M., Abdelraheem, A. M., Abdel-Rahim, M. A. & Moustafa, S. Substituting silver for tellurium in selenium-tellurium thin films for improving the optical characteristics. *J. Inorg. Organomet. Polym. Mater.* **32**, 2009. 10.1007/s10904-022-02250-y (2022).

[58] El-Mallah, H., Gaml, E. A., Menshawy, S. & El-Said, D. Morphological and optical properties induced by ultraviolet radiation of a thiadiazole derivative. *J. Mater. Sci. Mater. El.* **32**, 5381. 10.1007/s10854-021-05261-6 (2021).

[59] Elesh, E., Abul-Nasr, K. T., Abdelghany, A. & El-Damhogi, D. Thermal annealing enhanced morphological, nonlinear characteristic, and optical features of Victoria blue nanofilms for photonic application. *Optik* **295**, 171486. 10.1016/j.ijleo.2023.171486 (2023).

[60] Jrad, A., Naffouti, W., Ben Nasr, T., Ammar, S. & Turki-Kamoun, N. Effect of manganese concentration on physical properties of ZnS: Mn thin films prepared by chemical bath deposition. *J. Mater. Sci. Mater. El.* **28**, 1463. 10.1007/s10854-016-5682-z (2017).

[61] Yadav, S. K., Atyia, H., Fouad, S., Sharma, A. & Mehta, N. 2D/2D porous Co_3_O_4/_rGO nanosheets act as an electrochemical sensor for voltammetric tryptophan detection. *Diam. Relat. Mater.* **136**, 110030. 10.1016/j.diamond.2023.110030 (2023).

[62] Alibwaini, Y. et al. Synthesis, characterizations, optical and photoluminescence properties of polymer blend PVA/PEG films doped eosin Y (EY) dye. *Opt. Mater.* **111**, 110600. 10.1016/j.optmat.2020.110600 (2021).

[63] Spitzer, W. & Fan, H. Determination of optical constants and carrier effective mass of semiconductors. *Phys. Rev.* **106**, 882. 10.1103/PhysRev.106.882 (1957).

[64] Tian, S. et al. Optimization of tail state Urbach energy enables efficient organic solar cells and perovskite/organic tandem solar cells. *Org. Electron.* **113**, 106714. 10.1016/j.orgel.2022.106714 (2023).

[65] Landi, I. R., Segundo, E., Freitas, M., Vasilevskiy, J. & Carneiro, C.J. Tavares. Use and misuse of the Kubelka-Munk function to obtain the band gap energy from diffuse reflectance measurements. *Solid State Commun.* **341**, 114573. 10.1016/j.ssc.2021.114573 (2022).

[66] Chithambararaj, A. & Bose, A. C. Hydrothermal synthesis of hexagonal and orthorhombic MoO_3_ nanoparticles. *J. Alloy. Compd.* **509**, 8105. 10.1016/j.jallcom.2011.05.067 (2011).

[67] Udvardi, B. et al. Effects of particle size on the attenuated total reflection spectrum of minerals. *Appl. Spectrosc.* **71**, 1157. 10.1177/0003702816670914 (2017). 27671141 10.1177/0003702816670914

[68] Rouhi, H. F., Aghaei, F., Kalangestani, F. C., Chenari, H. M. & Nilkar, M. Enhanced electrocatalytic properties of plasma-treated MoS_2_-NiO-PVA nanofibers for hydrogen evolution reaction: A study of surface modifications and charge transfer kinetics. *Appl. Surf. Sci.*10.1016/j.apsusc.2025.163274 (2025).

[69] Carbone, M., Missori, M., Micheli, L., Tagliatesta, P. & Bauer, E. M. NiO pseudocapacitance and optical properties: Does the shape win?. *Materials* **13**, 1417. 10.3390/ma13061417 (2020). 32245018 10.3390/ma13061417 PMC7142826

[70] Tian, K., Wei, L., Zhang, X., Jin, Y. & Guo, X. Membranes of carbon nanofibers with embedded MoO3 nanoparticles showing superior cycling performance for all-solid-state flexible supercapacitors. *Mater. Today Energy* **6**, 27. 10.1016/j.mtener.2017.08.004 (2017).

[71] Kalangestani, F. C., Aghaei, F., Shahidani, HS., Nourmohammadian, M. Hydrothermal Synthesis of MSe_2_ (M = Mn, Ni) on the MoSe_2_@MWCNT Composite: Advancing Supercapacitor Electrode Efficiency, *ACS Appl. Electron. Mater.* **7**(10), 4649–4661. 10.1021/acsaelm.5c00609 (2025).

[72] Rani, S. et al. MoS_2_ nanoflower and cysteine-conjugated AgNPs based electrochemical biosensor for detection of NS1 protein specific to dengue virus. *J. Inorg. Organomet. Polym. Mater.* **34**, 4985–4995. 10.1007/s10904-024-03130-3 (2024).

[73] Ko, J. S. et al. High-rate capability of Na_2_FePO_4_F nanoparticles by enhancing surface carbon functionality for Na-ion batteries. *J. Mater. Chem. A* **5**, 18707. 10.1039/C7TA05680J (2017).

[74] Schoetz, T. et al. Disentangling faradaic, pseudocapacitive, and capacitive charge storage: A tutorial for the characterization of batteries, supercapacitors, and hybrid systems. *Electrochim. Acta* **412**, 140072. 10.1016/j.electacta.2022.140072 (2022).

[75] Dunn, A. Determination of an absolute scale of capacitance. *Can. J. Phys.* **42**, 53. 10.1139/p64-005 (1964).

[76] Ning, J. et al. Superior pseudocapacitive storage of a novel Ni3Si2/NiOOH/graphene nanostructure for an all-solid-state supercapacitor. *NanoMicro. Lett.* **13**, 1. 10.1007/s40820-020-00527-w (2021). 10.1007/s40820-020-00527-w PMC8187555 34138217

[77] Bayatpour, S., Afsharpour, M., Dini, Z. & Naderi, H. R. High performance supercapacitor electrodes using functionalized CNTs/MoO_3_ with natural polysaccharide binders. *J. Mater. Sci. Mater. El* **31**, 6150. 10.1007/s10854-020-03168-2 (2020).

[78] Zhao, N. et al. Simple electrodeposition of MoO_3_ film on carbon cloth for high-performance aqueous symmetric supercapacitors. *Chem. Eng. J.* **390**, 124477. 10.1016/j.cej.2020.124477 (2020).

[79] Wang, S. et al. Electrochemical impedance spectroscopy. *Nat. Rev. Methods Primers* **1**, 41. 10.1038/s43586-021-00039-w (2021).

[80] Godlaveeti, S. K. et al. Boosted electrochemical performance of SnO_2_/Mn_3_O_4_-Mn_2_O_3_ composite supported on reduced graphene oxide for supercapacitor applications. *Int. J. Hydrogen Energy* **102**, 1399–1410. 10.1016/j.ijhydene.2025.01.129 (2025).

[81] Rouhi, H. F., Yıldırım, F., Chenari, H. M., Biber, M. & Aydoğan, S. Highly air-stable and sensitive self-powered broadband photodetector based on a PVA–MoS_2_ fibers/n-Si heterojunction. *ACS Appl. Electron. Mater.* **9**, 757–765. 10.1021/acsaelm.4c01854 (2025).

[82] Patil, A. R. et al. Synthesis, analysis, and characterizations of microspherical MoO_3_ thin films for energy storage. *J. Mater. Sci. Mater. El* **35**, 590. 10.1007/s10854-024-12361-6 (2024).

[83] Chen, Y. et al. ShenCyanide-metal framework derived porous MoO_3_-Fe_2_O_3_ hybrid micro- octahedrons as superior anode for lithium-ion batteries. *Chem. Eng. J.* **426**, 130347. 10.1016/j.cej.2021.130347 (2021).

[84] Kalangestani, F. C., Simiari, M. & Ghodsi, F. E. Fabrication of V_2_O_5_/NiO double-layer film with hydrophilic property and evaluation of the effects of V_2_O_5_ coating on electro-optical parameters of NiO film. *Appl. Phys. A* **128**, 395. 10.1007/s00339-022-05545-6 (2022).

[85] Wang, X. et al. Fabrication of superhydrophilic self-cleaning SiO_2_–TiO_2_ coating and its photocatalytic performance. *Ceram. Int.* **48**, 20033. 10.1016/j.ceramint.2022.03.278 (2022).

[86] Nundy, S., Ghosh, A. & Mallick, T. K. Hydrophilic and superhydrophilic self-cleaning coatings by morphologically varying ZnO microstructures for photovoltaic and glazing applications. *ACS Omega* **5**, 1033. 10.1021/acsomega.9b02758 (2020). 31984259 10.1021/acsomega.9b02758 PMC6977091

[87] Zhao, W. & Lu, H. Self-cleaning performance of super-hydrophilic coatings for dust deposition reduction on solar photovoltaic cells. *Coatings* **11**, 1059. 10.3390/coatings11091059 (2021).

[88] Wang, X., Yang, L., Yang, D.-Q. & Sacher, E. Surface wettability effects on self-cleaning efficacy: Outdoor experimental study. *Sol. Energy* **266**, 112190. 10.1016/j.solener.2023.112190 (2023).

[89] Fromel, M. et al. Superhydrophilic polymer brushes with high durability and anti-fogging activity. *ACS Appl. Polym. Mater.* **3**, 5291. 10.1021/acsapm.1c01090 (2021).

[90] Ke, C., Zhang, C., Chen, H. & Jiang, Y. Robust superhydrophilic antifogging coatings by a facile sol–gel method. *J. Coat. Tech. Res.* **20**, 1. 10.1007/s11998-022-00748-1 (2023).

[91] Wang, X. et al. A multifunctional and environmentally-friendly method to fabricate superhydrophilic and self-healing coatings for sustainable antifogging. *Chem. Eng. J.* **409**, 128228. 10.1016/j.cej.2020.128228 (2021).

[92] Mansoor, B. et al. Polyvinyl alcohol (PVA) based super-hydrophilic anti-fogging layer assisted by plasma spraying for low density polyethylene (LDPE) greenhouse films. *Prog. Org. Coat.* **159**, 106412. 10.1016/j.porgcoat.2021.106412 (2021).

[93] Wang, X., Nshimiyimana, J. P., Huang, D., Diao, X. & Zhang, N. Durable superhydrophilic and antireflective coating for high-performance anti-dust photovoltaic systems. *Appl. Nanosci.* **11**, 875. 10.1007/s13204-020-01643-0 (2021).

[94] Hossain, M. I. et al. Hydrophilic antireflection and antidust silica coatings. *ACS Omega* **6**, 5276. 10.1021/acsomega.0c05405 (2021). 33681568 10.1021/acsomega.0c05405 PMC7931203

[95] Yuan, J., Yan, S. & Zhang, X. Superhydrophilic antifogging broadband antireflective coatings with worm-like nanostructures fabricated by one dip-coating method and calcination. *Appl. Surf. Sci.* **506**, 144795. 10.1016/j.apsusc.2019.144795 (2020).

[96] Mohammed, G., El-Sayed, A. M. & El-Gama, S. Effect of M nitrates on the optical, dielectric relaxation and porosity of PVC/PMMA membranes (M = Cd Co, Cr or Mg). *J. Inorg. Organomet. Polym. Mater.* **30**, 1306. 10.1007/s10904-019-01307-9 (2020).

[97] Cassie, A. & Baxter, S. Wettability of porous surfaces. *Trans. Faraday Soc.*10.1039/TF9444000546 (1944).

